# Plant immunity in natural populations and agricultural fields: Low presence of pathogenesis-related proteins in *Solanum* leaves

**DOI:** 10.1371/journal.pone.0207253

**Published:** 2018-11-09

**Authors:** Åsa Lankinen, Kibrom B. Abreha, Laura Masini, Ashfaq Ali, Svante Resjö, Erik Andreasson

**Affiliations:** Plant Protection Biology, Swedish University of Agricultural Sciences, Alnarp, Sweden; Agriculture and Agri-Food Canada, CANADA

## Abstract

Plant immunity has mainly been studied under controlled conditions, limiting our knowledge regarding the regulation of immunity under natural conditions where plants grow in association with multiple microorganisms. Plant pathology theory, based on laboratory data, predicts complex biochemical plant-pathogen interactions leading to coevolution of pathogen infectivity vs. plant recognition of microbes in multiple layers over time. However, plant immunity is currently not evaluated in relation to ecological time-scales and field conditions. Here we report status of immunity in plants without visible disease symptoms in wild populations of nightshades, *Solanum dulcamara* and *Solanum nigrum*, and in agricultural fields of potato, *Solanum tuberosum*. We analysed presence of pathogenesis-related proteins in over 500 asymptomatic leaf samples collected in the field in June, July and August over three years. Pathogenesis-related proteins were present in only one-third of the collected samples, suggesting low activity of the immune system. We could also detect an increase in pathogenesis-related proteins later in the growing season, particularly in *S*. *tuberosum*. Our findings, based on pathogenesis-related protein markers, indicate major gaps in our knowledge regarding the status and regulation of plant immunity under field conditions.

## Introduction

Over the past decades, laboratory studies of plant-pathogen interactions have uncovered several molecular components of the plant immune system. Induced responses of the plant immune system involves recognition of microbes and subsequent activation of a complex signalling and response pathway acting at different time points. Some of the most studied responses of immunity involve pathogenesis-related (PR) proteins. Plant non-self-recognition mechanisms are proposed to initiate two different levels of induced responses when microbes are detected [1]. The first level of immunity (PAMP-triggered immunity, PTI) responds to conserved pathogen-associated molecular patterns (PAMPs) on the cell surface and results in changes that in the end eradicate the non-successful pathogen or limit its spread through the plant. These changes include the activation of MAP kinase signalling cascades, expression of genes encoding PR proteins, and callose deposition [2]. The second level of immunity (effector-triggered immunity, ETI) involves pathogen recognition by intracellular receptors and transcriptional activation of, for example, PR proteins [3,4]. Salicylic (SA) and jasmonic acid (JA) play a crucial role in immune signalling pathways and the induction of PR proteins. PR1, for example, is a classic SA marker with an antimicrobial function while PR2 and 3 are more closely related to JA and are possibly associated with insect attack [5–7]. Genetic and biochemical evidence suggest that PR1 binds sterols [8]. Sterols in the host are known to be necessary for growth of sterol-auxotroph organisms such as oomycete pathogens, e.g. *Phytophthora infestans*, causing potato late blight. PR2 and 3 are glucanases and chitinases, which are involved in degradation of cell wall components of the enemies [7]. Thus, all these proteins are important components of plant immunity.

The two levels of immunity of plant pathology theory states coevolution of pathogen infectivity vs. plant recognition of microbes, where PTI is a general defence evolving first and later on ETI evolves towards specialized pathogens [1,9]. However, PAMPs and effectors are not always possible to distinguish, and PTI and ETI often share common genes, pathway and expression patterns [3,10,11]. Moreover, there is a need to evaluate this theory in relation to ecological time-scales and field conditions [11,12]. In contrast to laboratory conditions, plants under field conditions interact with multiple microorganisms, including bacteria, fungi and oomycetes (plant microbiota, [13]). The prediction that PAMPs activate a defence response, and that only disease down-regulates this immune response (effector-triggered susceptibility, ETS) can be followed by an expectation that PTI should be constantly activated in the field in the presence of PAMPs, even without a successful pathogen attack. However, ecological and epidemiological studies from wild study systems suggest large variation in pathogen occurrence and prevalence between years and populations [14,15], potentially indicating less frequent immunity activation under field conditions. To date, no study using molecular markers of immunity activation based on a large number of samples of field material has been carried out.

To increase our understanding of plant immunity under field conditions, we investigated the actual status of immune responses in both natural populations (wild Bittersweet nightshade, *Solanum dulcamara* and European black nightshade, *Solanum nigrum*) and in agricultural settings (cultivated potato, *Solanum tuberosum*). Comparison of crops and their wild relatives is of interest because breeding or cultivation may alter plant defence [16]. We analysed over 500 apoplastic leaf samples for the presence of PR proteins (PR1 and PR2+3), as markers for plant immunity. Evidence from previous studies show that all these three species accumulate PR proteins in the apoplast after biotic stress in controlled conditions as shown by 1D-SDS PAGE analysis [6,17–19]. We therefore collected such samples directly in the field from asymptomatic plants during three consecutive years in the three summer months in southern Sweden (S1 Table, [20]). The reason for sampling symptomless plant material was that we were mainly interested in immunity activation in the presence of PAMPs, rather than down-regulation of immunity caused by disease, ETS. We hypothesized higher levels of PAMPs and consequently more frequent PTI activation, later in the season because the density of microorganisms could be expected to become higher with the increase in temperature and precipitation over the summer in Sweden [21]. This is particularly likely for potato fields where several diseases, e.g. early and late blight, are known to become a problem later in the season [22,23]. Because our tested potato clones differed in presence of resistance (R) genes to infection by the pathogen *P*. *infestans*, causing late blight, we tested if resistant vs. susceptible clones differed in immunity activation over the season.

In this study, we focused on PR proteins rather than gene expression because we expected PR proteins to be more stable and more closely represent the visible phenotype. We also compared our data on PR proteins to a small published data set on gene expression of PR1 and PR2 in cultivated potato fields [24]. Our overall aim was to investigate the status of plant immunity under natural conditions, as a first step towards developing a more complete understanding of plant immunity outside of the laboratory. By sampling and extracting PR proteins directly in the field, we were able to get an indication of this component of immunity activation in agricultural fields and natural populations.

## Materials and methods

### Plant material

For *S*. *tuberosum*, we sampled six different potato clones at four agricultural field sites in southern Sweden (S1 Table). We used five of these clones to compare the relation between immunity activation and presence of R genes to *P*. *infestans* based on resistance reactions in controlled experiments [18,19]. We sampled three of the sites over multiple years. Sampled fields were unsprayed except one site (S1 Table) from where we collected plant material from both unsprayed plants and plants sprayed with the inducing agents β-aminobutyric acid and phosphite (2011) or fungicide (2012). We pooled sprayed and unsprayed samples collected at the same site as these samples did not differ in presence of PR proteins (2011: PR1 χ^2^ = 0.20, df = 1, *P* = 0.66; PR2+3 χ^2^ = 2.23, df = 1, *P* = 0.13; 2012: PR1 χ^2^ = 0, df = 1, *P* = 1; PR2+3 χ^2^ = 0.13, df = 1, *P* = 0.72). On average, we analysed 4.3 biological replicates per clone and sampling event (total *N* = 225).

For the wild species, we sampled natural populations (*S*. *nigrum*: *N* = 3, *S*. *dulcamara*: *N* = 8), with two and six of these populations, respectively, sampled repeatedly in multiple years (S1 Table). In 2011, we also included samples from plants originating from these populations but grown in an agricultural field at the Alnarp experimental garden (*S*. *nigrum*: *N* = 2 populations, *S*. *dulcamara*: *N* = 5 populations). *Solanum nigrum* was only sampled in July and August as this species germinates later in the season. On average, we analysed 5.9 and 6.2 biological replicates per sampling event in *S*. *nigrum* (total *N* = 53) and *S*. *dulcamara* (total *N* = 294), respectively.

### Apoplast sample preparation in the field

We followed Andreasson et al. [25] for apoplast isolation from 4–5 relatively young, fully expanded leaves of all species, but performed all sample preparation immediately at the field sites in the back of a small van [20]. The age, size and position of the leaves was similar at each sampling month. We directly froze aliquots with a protease inhibitor mix (Sigma) in liquid nitrogen and stored them at -80°C until SDS-PAGE separation. Field sampling took place between 10 am and 3 pm during days of sunny or overcast weather (no rain).

### SDS-PAGE separation and identification of PR proteins

We denatured proteins by dissolving the samples in 2X SDS-PAGE protein loading buffer with DTT [25]. We loaded 30 μl of the sample containing linearized protein onto the gel and separated for 6 cm with 13% SDS-PAGE. We determined the presence (1) or absence (0) of PR1 and the combination of PR2 and PR3 (PR2+3) in the samples from the bands on the Coomassie blue stained SDS-PAGE images [26] using the known molecular mass for these proteins. Samples identified to be contaminated with rubisco, from inspection of the gels, were removed from all analyses. Pictures of gels are available as supporting information (S1–S3 Datasets). The loaded material on each lane corresponds to apoplastic fluid from ca. 0.4 leaves. To confirm the consistency of our classifications of the gels, two independent people investigated 68 samples (randomly chosen with respect to species and sampling time in 2010 and 2011). These samples confirmed 100% repeatability of detection of the presence vs. absence of PR proteins.

We verified the identity of the bands by trypsin digestion and mass spectrometry essentially as described previously by Ali et al. [18]. Briefly, the peptides were separated in a 0.1% FA buffer using a 45 min linear gradient from 5% to 35% CAN using an Eksigent nanoLC2D HPLC system. The eluted peptides were analysed online using an LTQ Orbitrap XL ETD. The Orbitrap was operated in data dependent mode to automatically perform Orbitrap-MS and LTQ-MS/MS analysis. The four most intense ions were selected for fragmentation in the LTQ. The raw data from the Orbitrap was converted to Mascot Generic Format (MGF) and mzML [27] using ProteoWizard (version 3.0.11252) [28]. The Proteios software environment (2.20 dev build 4631) [29] was used to search the MGF files with Mascot (version 2.4.1) against a database consisting of all *S*. *tuberosum* proteins in UniProt as of 2017-09-22, concatenated with an equal size decoy database. Trypsin (one missed cleavage allowed) was used to generate peptides with search tolerances of 7 ppm for MS and 0.5 Da for MS/MS. Carbamidomethylation of cysteine residues was selected as a fixed modification and oxidation of methionine residues was selected as a variable modification. Proteios was used to calculate q values as described by Käll et al. [30]. The search results were then filtered at a q-value of 0.01. As PR2 and PR3 peptides were found in the same band they were combined in the analysis.

### Gel and RNA data analyses

We analysed incidence of PR proteins as presence relative to absence of PR1 and PR2+3 proteins using logistic regression in R [31]. Starting models investigated the effects of the categorical factors species/clone (depending on the analysis), sampling year, sampling month, and the interaction between species/clone and month. We also performed complementary analyses where we restricted the data (one or two months, two species or five potato clones) to obtain a balanced data set. We controlled for over-dispersion by refitting the model with quasibinomial errors. We assessed statistical significance (*P* < 0.05) by testing the change in deviance between successive models with an *F*-test. We excluded all non-significant factors or interactions using backwards deletion of higher-order interactions. We performed pairwise comparisons (Tukey) using the R package ‘multcomp’ [32].

To investigate whether the presence of both PR1 and PR2+3 in the same sample differed from that predicted from the frequencies of PR proteins alone, as an indication of co-expression, we used a Chi-square test of independence. We also used a Chi-square test of independence to analyse differences in co-expression between wild and cultivated species, where data for *S*. *dulcamara* and *S*. *nigrum* were pooled together to increase sample size. We used Yate’s correction for small expected values [33]. Samples where one of the PR proteins could not be identified as either present or absent (*N* = 4) where excluded from the analysis.

For the comparison of our data with a published RNA seq data from potato fields in Germany (*N* = 24) [24], we determined presence and absence of gene expression of PR1 (PGSC0003DMG400005111, cut off value of 10 fragments per kilobase of exon per million fragments mapped (FPKM) data) and PR2 (PGSC0003DMG402010492, cut off value of 20 FPKM data).

## Results

**Fig 1:**
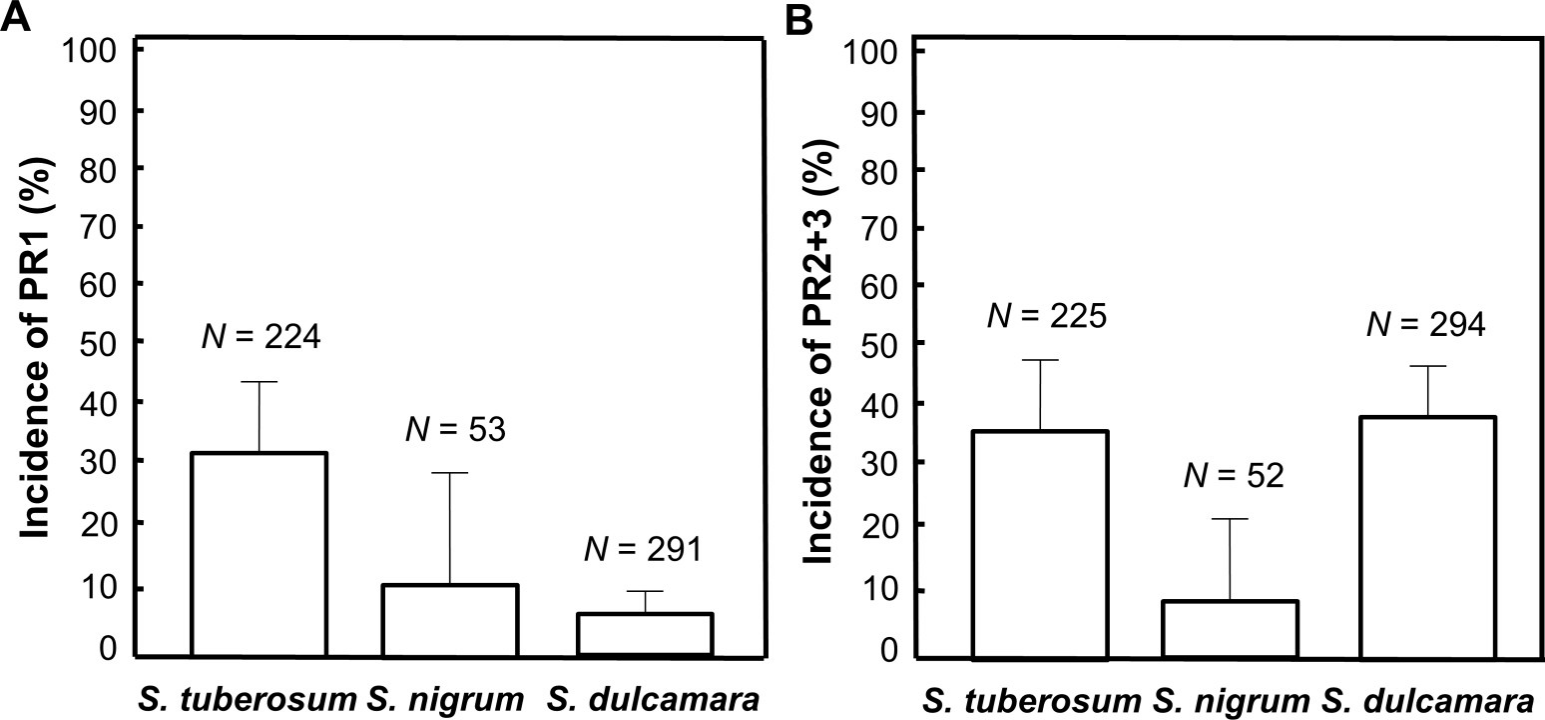
Incidence of PR proteins in cultivated *Solanum tuberosum*, and wild *Solanum nigrum* and *Solanum dulcamara*. Mean ± 95% CI of (A) PR1 and (B) PR2+3. Sampling was conducted in the field over three years during the three summer months per year. *N* = number of apoplastic samples.

Analysis of apoplastic samples of the three species from all three investigated years showed that PR proteins (of either type) were present in 36.4% (208 out of 571) of all samples (Fig 1A and 1B). We analysed the PR protein identity of the bands by mass spectrometry analysis and identified 24 peptides from PR1 (2 different proteins) in the low molecular band that we classified as PR1, and 55 peptides from PR2 and PR3 (6–9 different proteins) from the higher molecular bands that were classified as PR2+3 (S2 Table). In our samples, PR1 was present in 16.5% and PR2+3 in 32.7% (Fig 1A and 1B), indicating that neither PR1 nor PR2+3 were abundant at a high frequency. Our analysis of the published RNA seq data from cultivated potato fields in Germany indicated a similar frequency of gene expression (PR1 17% and PR2 25%, *N* = 24).

**Fig 2:**
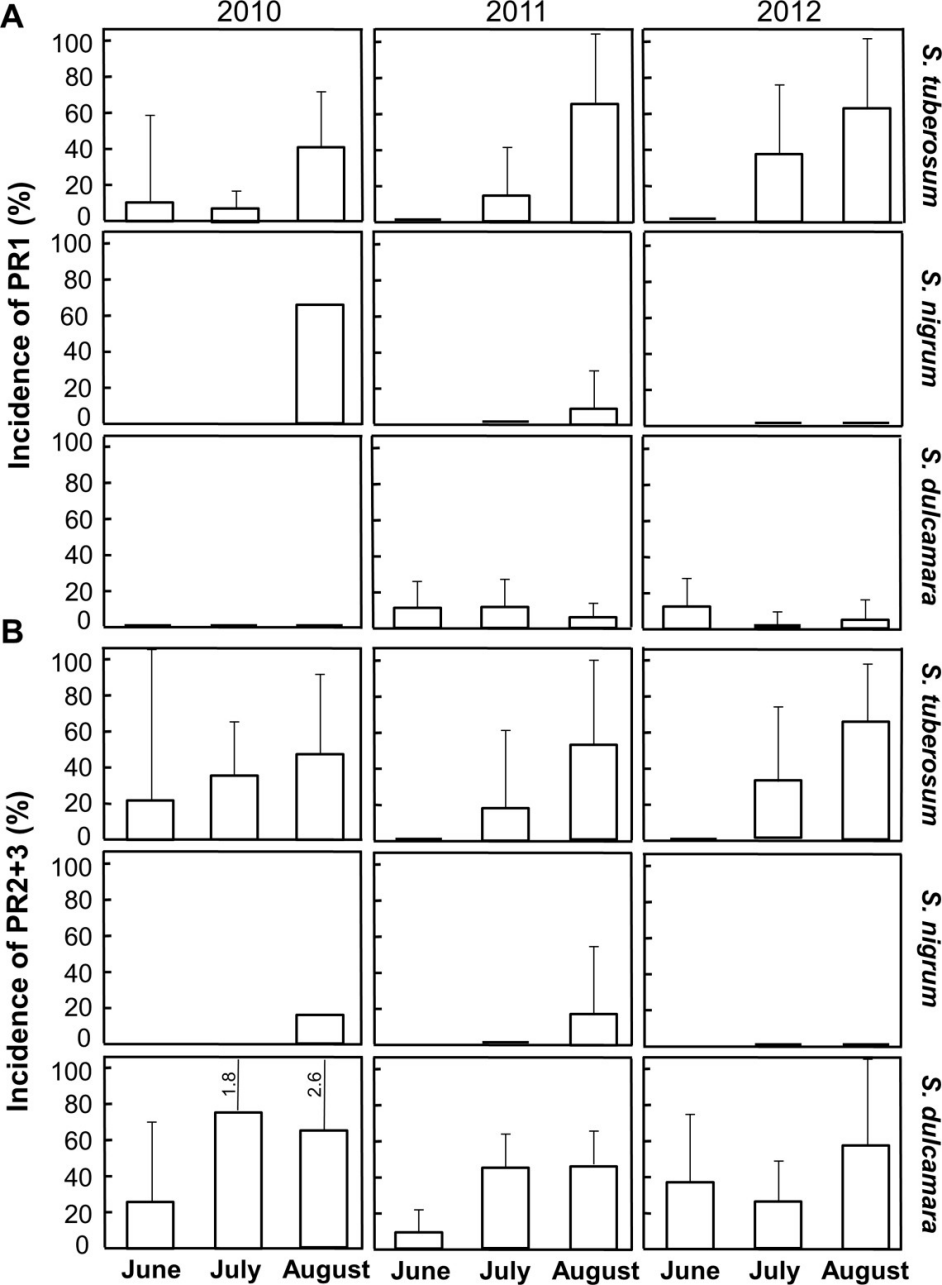
Incidence of PR proteins per species and month in cultivated *Solanum tuberosum*, and wild *Solanum nigrum* and *Solanum dulcamara*. Mean ± 95% CI of (A) PR1 and (B) PR2+3. Sampling was conducted in the field over three years during the three summer months per year. Numbers indicate the maximum value of large error bars. Statistical test results are given in the text. See S1 Table for a list of field locations.

The presence of PR1 protein in the three species during the two months when they were all sampled (July and August) differed among species and showed a general increase with month (Species: *F*_2,78_ = 14.3, *P* < 0.001; Month: *F*_1,78_ = 9.75, *P* = 0.002, Fig 2A), but was unaffected by year (*P* = 0.23). PR1 was significantly less abundant in *S*. *dulcamara* than in *S*. *tuberosum* (*P* < 0.001) and *S*. *nigrum* (*P* = 0.016). Comparison of only *S*. *tuberosum* and *S*. *dulcamara* during June, July, and August (because *S*. *nigrum* was not sampled in June) confirmed the difference in PR1 presence between species and indicated that the increase at the end of the season was only significant for *S*. *tuberosum* (Species × Month: *F*_2,91_ = 7.75, *P* = 0.0008, Fig 2A). Moreover, year had an effect (*F*_2,91_ = 4.05, *P* = 0.021), but the higher frequency of PR1 in 2011 compared to in 2010 was only marginally significant (*P* = 0.041).

The presence of PR2+3 did not show the same pattern across species as PR1; across all three species, PR2+3 was less frequently found in *S*. *nigrum* than in the other two species (*F*_2,79_ = 4.64, *P* = 0.012; *S*. *tuberosum*: *P* = 0.017; *S*. *dulcamara*: *P* = 0.021, Fig 2B). Analysing only *S*. *tuberosum* and *S*. *dulcamara* during June, July, and August revealed an effect of month (*F*_2,96_ = 7.99, *P* = 0.0006), with increased PR2+3 abundance in July (*P* = 0.019) and August (*P* < 0.001) compared to June. Thus, our expectation of more frequent PTI activation later in the season was consistent with the higher presence of PR2+3 for both *S*. *tuberosum* and *S*. *dulcamara* later in the season, while the monthly increase in PR1 was only significant in *S*. *tuberosum*.

Co-occurrence of both PR1 and PR2+3 proteins in the same sample was more common than that predicted from the frequencies of the PR proteins alone in all three species (χ^2^ = 8.01–79.2, df = 1, *P* < 0.0046), suggesting co-expression. Co-expression occurred more frequently in *S*. *tuberosum* (13% of all samples) than in the two wild species (4–6%) (χ^2^ = 11.4, df = 1, *P* < 0.001). In *S*. *tuberosum*, co-expression in samples where at least one PR protein was present was 50% in June, 48% in July and 62% in August (pooled for the three years). In *S*. *dulcamara*, co-expression was 23% in June, 18% in July and 11% in August.

**Fig 3:**
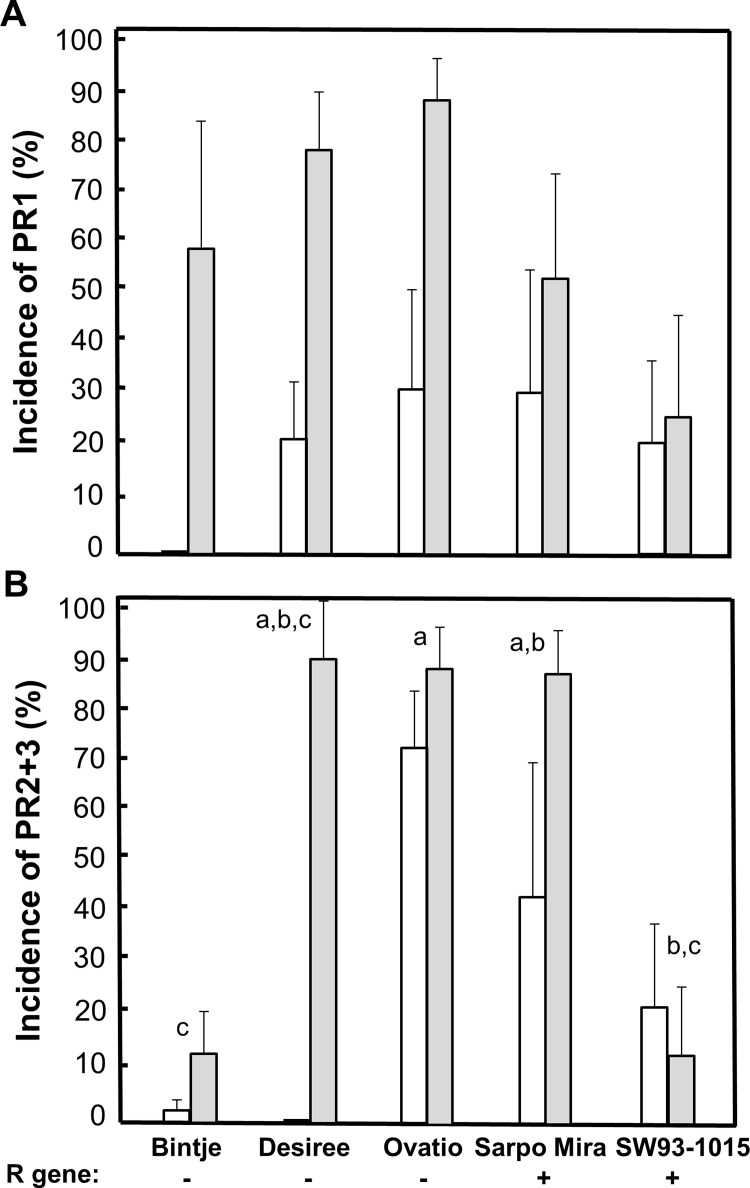
Incidence of PR proteins in five clones of cultivated *Solanum tuberosum*. Mean ± 95% CI of (A) PR1 and (B) PR2+3. Sampling was conducted in the field over three years during the two later summer months per year. White bars = July sampling, gray bars = August sampling. R gene -/+ = absence vs. presence of resistance (R) gene with known resistance reactions to infection by *Phytophthora infestans*. Different letters denote significant (*P* < 0.05) difference between clones (pooled for July and August). No significant pairwise differences were found between clones for PR1. See S1 Table for a list of field locations.

We investigated whether PR proteins were more frequent late in the season in potato clones with resistance (R) genes to *Phytophthora infestans* inoculation compared to in clones without R genes, as the occurrence of potato diseases such as late blight caused by *P*. *infestans* are known to increase over the season. Among the five investigated potato clones, PR1 presence was higher in August than in July (*F*_1,34_ = 12.5, *P* = 0.0012), and we detected an effect of clone (*F*_4,34_ = 2.96, *P* = 0.034) and year (*F*_2,34_ = 3.40, *P* = 0.045). However, no pairwise clone comparisons were significant for PR1 (*P* > 0.056, Fig 3A). PR2+3 presence was higher later in the season (*F*_1,36_ = 7.87, *P* = 0.008) and differenced among clones (*F*_4,36_ = 11.2, *P* < 0.0001), also showing some pairwise differences (Fig 3B). Despite these clonal differences, there was no significant difference in PR presence in relation to occurrence of R genes in either July or August (*F*_1,19_ = 2.63, *P* = 0.12, Fig 3A and 3B).

## Discussion

In this study we showed that PR1 and PR2+3 proteins, as classical markers for major plant immunity pathways, are present in only one third of over 500 samples of plant material collected under natural conditions in wild nightshade populations and in cultivated potato fields during three years in south Sweden (Fig 1). Basically no pathogen-derived symptoms were visible in our wild populations and potato fields, apart from later in the season in the potato fields where late blight disease started to emerge occasionally in the area. We also found a similar low degree of immune activation when analysing a small published data set on gene expression of PR1 and PR2 in potato from agricultural fields in Germany [24]. This low proportion of immunity activation contrasts somewhat with a prediction from plant pathology theory based on laboratory data that suggests that PTI should be very frequently expressed if plants are not showing clear disease symptoms (ETS) in the presence of PAMPs, i.e. ETS is the main down regulatory mechanisms for immunity [1]. Interestingly, in contrast to our results and the results from Germany [24], a limited proteomic analysis of the apoplast in the short life cycle plant *Arabidopsis thaliana* indicated high abundances of defence- and stress-related proteins in the wild populations [34]. Given the large variation in pathogen incidence and prevalence indicated in wild plant populations [14,15], it would indeed be important to sample over several years and across multiple populations.

We hypothesized higher levels of PAMPs and consequently more frequent PTI activation later in the season because the density of microorganisms is expected to increase over the growing season in response to the change in weather condition occurring in Sweden and the increase in potato diseases over the summer. While we have not been able to find a study that measured absolute number of microbes or PAMPs on plants over time, it has been shown that the leaf microbiome changes in diversity over the growing season [35]. As hypothesized, our results suggested an increase in PR proteins later in the summer, particularly in potato where both PR1 and PR2+3 increased (Fig 2). We cannot exclude other possible explanations for the higher frequency of PR proteins later in the season, such as the fact that plants were older, and started flowering and set seeds [36]. However, as we used a similar age of all leaves across the sampling months, it is unlikely that leaves were exposed to microbes for a longer period of time later in the season.

The higher presence of PR2+3 relative to PR1 in *S*. *dulcamara* compared with potato (Fig 2) could be related to differences in the immunity response between species or by less favourable conditions for PR1 production in wild populations due to greater insect damage, favouring induction of JA [5,37]. Because the wild populations often grow close to or in agricultural fields we anticipate that these plants will be exposed to similar microorganism populations as in the agricultural landscape. However, during sampling of *S*. *dulcamara*, we sometimes encountered plants with leaves showing signs of insect attack, e.g. by *Phyllotreta* spp. Interestingly, *S*. *dulcamara* is known to be attacked by several insect species [38] and induced defence to herbivory has been documented to have a negative effect on insects, lasting for up to 40 days [39,40]. Another difference between the investigated species was the more frequent co-activation of induced responses through SA and JA signalling pathways in potato compared to in the wild species (Fig 2). This result may reflect the generally low incidence of PR1 expression in *S*. *dulcamara*. It should be noted that co-expression in *S*. *tuberosum* over the growing season was only found in around 50% of all samples with at least one PR protein present. The results on co-expression indicate a low degree of co-regulation in line with the classical association to different hormone signalling pathways. Even though there were no major differences between cultivated potato and natural *Solanum* populations for both PR proteins, in cultivated potato, PR1 appeared more frequent at the end of the season and co-expression of PR1 and PR2+2 was higher. These differences may be species-specific or due to changes in defence mechanisms caused by domestication [16]. Our data does not, however, support the loss of general defence mechanisms during breeding, as previously suggested [41]. The species differences should be investigated further in studies disentangling the immune response and the exposure to pests and pathogens.

Thomma et al. [3] suggested that PTI and ETI should be regarded as two extremes of a continuum, as it is not always possible to separate PAMPs and effectors. In our investigated potato clones with presence vs. absence of resistance (R) genes towards infection by *P*. *infestans*, we hypothesized that clones with R genes would express ETI towards the end of the growing season when it is well known that potato fields starts to be infected by *P*. *infestans* in south Sweden [42]. Thus, in line with plant pathology theory, we expected higher levels of immunity activation in clones with R genes. We found no clear support for this hypothesis (Fig 3), potentially indicating a lack of difference in PR protein expression between PTI and ETI [3,10,11]. To fully interpret these results, we would need to estimate *P*. *infestans* exposure in the field as well as the occurrence of other microbes/PAMPs at the time of sampling.

The low frequency of PR1 and PR2+3 proteins detected in plants of this study, despite a high anticipated frequency of PAMPs in natural and agricultural habitats during the summer in south Sweden, could indicate that plant immunity is difficult to activate under field conditions, e.g. high threshold levels of PAMPs are needed, or potential microbes have been adapted to reduce PAMPs, or, alternatively thresholds are sufficient for immune activation but plants have efficient mechanisms to repress immune responses because of the cost of expressing defences [43,44]. Theoretical work has suggested that a combination of the cost of having the resistance alleles, the cost of expressing resistance in the presence of pathogens, a heterogeneous environment and pathogen prevalence are important factors for evolution of plant resistance, and that prevalence of disease in most cases is not consistently high [45]. In line with this theoretical study, a recent study in *A*. *thaliana* highlighted that costs of possesing resistance alleles in the absence of disease may indeed be low [46]. We can hypothesize that if costs mostly are connected to expression of immunity, plants should benefit from harbouring resistance allelles but have the capacity to down-regulate immune responses, such as in the case when pathogens are too few to infect or cause any harm, or are of a non-virulent type or even a beneficial type [13,47]. There is evidence for immune repression occurring under controlled conditions mainly in *A*. *thaliana* [48]. Based on our findings, we propose that an important complement to current plant pathology theory would be to take immune repression and deactivation into account. In the future, it would also be of great importance to estimate additional components of plant immunity, which may be subjected to other fluctuations in time [13].

Induced responses could be a valuable trait in crops as not only pathogens but also chemical treatments such as β-aminobutyric acid can activate immunity, allowing reduced fungicide treatments [49]. Our finding that plant immunity is not always activated under field conditions, particularly early in the season, implies that there is scope for using plant resistance inducers (PRI), which can only work if immunity is not already induced, as a disease management strategy [50]. Our results suggest that application early in the season could have the greatest chance of success [49], and point to that different background activation of immunity may be a possible explanation for why the efficiency of PRIs under field conditions vary between 4 to 90% [51]. Other factors should also be taken into consideration for the timing of application of PRIs, e.g. costs of immunity activation could vary over the season leading to yield penalties.

In conclusion, PR proteins were present in only one-third of all samples in both natural populations and in agricultural settings in this study of Solanaceae species. In future studies, it would be important to confirm our results of plant immunity status in the field with additional markers and study systems. While our data represents a snapshot in time, which does not necessarily constitute plant immunity reactions from an ecological and epidemiological viewpoint, our findings highlight the importance of considering ecological time-scales of plant-pathogen interactions and suggests major gaps in our knowledge regarding the regulation of plant immunity in the field. Another complement to recording plant immunity in the field would be to quantify microbes and PAMPs exposed to plants in natural and agricultural environments.

## Supporting information

S1 Table Location and collection time of secretome samples in one cultivated and two wild *Solanum* species. (DOCX)

S2 Table PR1 peptides and proteins identified in the low molecular band, and PR2 and PR3 peptides and proteins identified in the high molecular bands. (DOCX)

S1 Dataset Gel pictures PR proteins 2010. (PDF)

S2 Dataset Gel pictures PR proteins 2011. (PDF)

S3 Dataset Gel pictures PR proteins 2012. (PDF)
