# Supplementary material for: Plant immunity in natural populations and agricultural fields: Low presence of pathogenesis-related proteins in Solanum leaves
Source: PLoS One. 2018 Nov 9;13(11):e0207253. doi: 10.1371/journal.pone.0207253 (PMC6226184; doi:10.1371/journal.pone.0207253)
Supplement: S1 Table — (DOCX) [file pone.0207253.s001.docx]

**S1 Table.** **Location and collection time of secretome samples in one cultivated and two wild *Solanum* species**

| Clone | Site | 2010 | | | 2011 | | | 2012 | | | Habitat natural  populations |
| --- | --- | --- | --- | --- | --- | --- | --- | --- | --- | --- | --- |
|  |  | June | July | Aug | June | July | Aug | June | July | Aug |  |
| ***S. tuberosum*** **(cultivated species)** | | | | | | | | | | | |
| Bintje^1^ | Alnarp exp garden | x | x | x |  | x | x |  |  |  |  |
|  | Borgeby |  | x | x |  |  |  |  |  |  |  |
|  | Mosslunda |  | x |  | x^2^ | x^2^ | x^2^ | x^3^ | x^3^ | x^3^ |  |
| Desiree^1^ | Alnarp exp garden |  |  |  |  | x | x |  |  |  |  |
|  | Borgeby |  |  |  |  |  |  |  | x | x |  |
|  | Helgegården |  |  |  |  |  |  | x | x | x |  |
| Ovatio^1^ | Alnarp exp garden | x | x | x |  |  |  |  |  |  |  |
|  | Borgeby |  | x | x |  |  |  |  |  |  |  |
|  | Mosslunda |  | x |  | x^2^ | x^2^ | x^2^ | x^3^ | x^3^ | x^3^ |  |
| Superb | Alnarp exp garden | x |  |  |  |  |  |  |  |  |  |
| Sarpo Mira^1^ | Alnarp exp garden |  |  |  |  | x | x |  |  |  |  |
|  | Borgeby |  | x | x |  |  | x |  | x | x |  |
|  | Helgegården |  |  |  |  |  |  | x | x | x |  |
| SW93-1015^1^ | Alnarp exp garden |  |  |  |  | x | x |  |  |  |  |
|  | Borgeby |  | x | x |  |  | x |  | x | x |  |
|  | Helgegården |  |  |  |  |  |  | x | x | x |  |
| ***S. nigrum*** **(wild species)** | | | | | | | | | | | |
|  | Alnarp exp garden |  |  |  |  | x | x |  |  |  | agricultural field |
|  | Alnarp south |  |  | x |  |  | x |  |  |  | agricultural field |
|  | Borgeby |  |  |  |  |  | x |  | x | x | agricultural field |
|  | Spillepengen |  |  |  |  | x | x |  |  |  | disrupted wasteland |
| ***S. dulcamara*** **(wild species)** | | | | | | | | | | | |
|  | Alnarp exp garden |  |  |  |  | x | x |  |  |  | agricultural field |
|  | Alnarp hedge |  |  |  | x | x | x | x | x | x | in hedge along road |
|  | Alnarp pond | x |  |  | x |  | x |  |  |  | shaded pond |
|  | Lund genetikum | x | x | x | x | x | x | x | x | x | along parking space |
|  | Lomma 1 | x |  |  | x | x | x | x | x | x | wooded marsh |
|  | Lomma 2 | x | x | x | x | x | x | x | x | x | wooded along road |
|  | Lomma 3 |  |  |  | x | x | x |  | x | x | beach |
|  | Tvedöra |  |  |  | x | x | x |  |  |  | marsh |
|  | Spillepengen |  |  |  |  | x | x |  |  |  | disrupted wasteland |

^1^Potato clone used in comparison between immunity activation and presence of resistance (R) gene to *Phytophthora infestans*.

^2^Pooled data for samples from untreated plants and plants sprayed with BABA and phosphite.

^3^Pooled data for samples from untreated plants and plants sprayed with fungicide.
