# Supplementary material for: Plant immunity in natural populations and agricultural fields: Low presence of pathogenesis-related proteins in Solanum leaves
Source: PLoS One. 2018 Nov 9;13(11):e0207253. doi: 10.1371/journal.pone.0207253 (PMC6226184; doi:10.1371/journal.pone.0207253)
Supplement: S2 Table — (DOCX) [file pone.0207253.s002.docx]

**S2 Table. PR1 peptides and proteins identified in the low molecular band, and PR2 and PR3 peptides and proteins identified in the high molecular bands**

| Peptide | Uniprot id | PR protein |
| --- | --- | --- |
| LAAFAQNYANQR | M1A2A4 | PR1 |
| MQHSGGPYGENLAAAFPQLNAAGAVK | M1A2A4 | PR1 |
| QVGVGPMTWDNR | M1A2A4 | PR1 |
| QVGVGPMTWDNR Oxidation (M) | M1A2A4 | PR1 |
| VCGHYTQVVWR | M1A2A4 | PR1 |
| AAVQLWVGEKPNYNYGTNQCASGQVCGHYTQVVWR | Q941G6 | PR1 |
| AQNYANSR | Q941G6 | PR1 |
| AQVGVGPMSWDAGLASR | Q941G6 | PR1 |
| AQVGVGPMSWDAGLASR Oxidation (M) | Q941G6 | PR1 |
| AQVGVGPMSWDAGLASRAQNYANSR | Q941G6 | PR1 |
| AQVGVGPMSWDAGLASRAQNYANSR Oxidation (M) | Q941G6 | PR1 |
| CNNGWWFISCNYDPVGNWVGQRPY | Q941G6 | PR1 |
| GTGDFTGR | Q941G6 | PR1 |
| TGDCNLIHSGAGENLAK | Q941G6 | PR1 |
| FVGPAMENIYNALSSAGLQNQIK | M1APC7,M1APC8 | PR2 |
| FVGPAMENIYNALSSAGLQNQIK Oxidation (M) | M1APC7,M1APC8 | PR2 |
| NIEIILDVPNQDLEALANPSNANGWVQDNIR | M1APC7,M1APC8 | PR2 |
| HNLPLLANIYPYFAHADDNVPLSYALFK | M1APC7,M1APC8 | PR2 |
| IANNLPSDQDVIK | M1APC7,M1APC8 | PR2 |
| LGGQNIEIIVSESGWPSEGHPSATLENAR | M1APC7,M1APC8 | PR2 |
| LYNANNIK | M1APC7,M1APC8 | PR2 |
| LYNANNIKK | M1APC7,M1APC8 | PR2 |
| QQGLNDAGYQNLFDALVDSMYFATEK | M1APC7,M1APC8 | PR2 |
| QQGLNDAGYQNLFDALVDSMYFATEK Oxidation (M) | M1APC7,M1APC8 | PR2 |
| SFINPIIGFLAR | M1APC7,M1APC8 | PR2 |
| VSTATYLGLLTNTYPPR | M1APC7,M1APC8 | PR2 |
| IYYPDTNVFNALK | M1APC7,M1APC8,M1APB3,M1APB4,M0ZQP9,M0ZQQ0,M0ZQQ6 | PR2 |
| NHFPDVK | M1APC7,M1APC8,M1APB3,M1APB4,M1APC9 | PR2 |
| DGKPSEQHFGLFYPDK | M1APC7,M1APC8,M1APC9 | PR2 |
| DGKPSEQHFGLFYPDKRPK | M1APC7,M1APC8,M1APC9 | PR2 |
| FKYIAVGNEVDPGR | M1APC7,M1APC8,M1APC9 | PR2 |
| KDGKPSEQHFGLFYPDK | M1APC7,M1APC8,M1APC9 | PR2 |
| YIAVGNEVDPGR | M1APC7,M1APC8,M1APC9 | PR2 |
| TIETYLFAMFDENR | M1APC7,M1APC8,M1APC9,M1APC4 | PR2 |
| TIETYLFAMFDENR Oxidation (M) | M1APC7,M1APC8,M1APC9,M1APC4 | PR2 |
| TIETYLFAMFDENRK | M1APC7,M1APC8,M1APC9,M1APC4 | PR2 |
| TIETYLFAMFDENRK Oxidation (M) | M1APC7,M1APC8,M1APC9,M1APC4 | PR2 |
| TYYTNLINHVK | M1APC7,M1APC8,M1APC9,M1APC4 | PR2 |
| DSIFREEYK | M1APC9 | PR2 |
| HNLPLLANIYPYFAHADDNVPLSYALFNQQGR | M1APC9 | PR2 |
| NDAGYQNLFDALVDSMYFATEK | M1APC9 | PR2 |
| NDAGYQNLFDALVDSMYFATEK Oxidation (M) | M1APC9 | PR2 |
| SFINPIIGFLSR | M1APC9 | PR2 |
| YAQFVGPAMENIYNALSSAGLQNQIK | M1APC9 | PR2 |
| YAQFVGPAMENIYNALSSAGLQNQIK Oxidation (M) | M1APC9 | PR2 |
| YIAVGNEVDPGRDSGK | M1APC9 | PR2 |
| AGQGIGVGQDLVNNPDLVATDPIISFK | M0ZMG2 | PR3 |
| ELFEQMLSFR | M0ZMG2 | PR3 |
| ELFEQMLSFR Oxidation (M) | M0ZMG2 | PR3 |
| EMAAFFGQTSHETNGGSAGTFTGGYCFVR Oxidation (M) | M0ZMG2 | PR3 |
| GFYTYDAFIAAANSFPAFGTTGDDTAR | M0ZMG2 | PR3 |
| GFYTYDAFIAAANSFPAFGTTGDDTARK | M0ZMG2 | PR3 |
| GPIQLTHQSNYER | M0ZMG2 | PR3 |
| QIDQSER | M0ZMG2 | PR3 |
| RYCGMLNVPTGENLDCNNQR | M0ZMG2 | PR3 |
| RYCGMLNVPTGENLDCNNQR Oxidation (M) | M0ZMG2 | PR3 |
| VPGYGVITNIINGGLECGMGQNTAVDSR | M0ZMG2 | PR3 |
| YCGMLNVPTGENLDCNNQR | M0ZMG2 | PR3 |
| YCGMLNVPTGENLDCNNQR Oxidation (M) | M0ZMG2 | PR3 |
| EGNQVGSGFYGR | M0ZMG3 | PR3 |
| EIAAFFGQTSHETTGGSLSADGPFAGGYCFLR | M0ZMG3 | PR3 |
| GPIQLTGQSNYDLAGQAIGQDLVNNPDLVATDATVSFK | M0ZMG3 | PR3 |
| QPGYGVITNIINGGIECGK | M0ZMG3 | PR3 |
| AINEDLLNNPYLVATDPVISFK | M1AH25 | PR3 |
| GPIQLTSNLNYGPFGR | M1AH25 | PR3 |
| NNFYSYNAFITAAR | M1AH25 | PR3 |
| SYPGFGTTGDITTR | M1AH25 | PR3 |
| AIGVDLLNNPDLVATDSIISFK | M1CJU2,P52404 | PR3 |
| WQPSGTDQAANR | M1CJU2,P52404 | PR3 |
