# Supplementary material for: Plant immunity in natural populations and agricultural fields: Low presence of pathogenesis-related proteins in Solanum leaves
Source: PLoS One. 2018 Nov 9;13(11):e0207253. doi: 10.1371/journal.pone.0207253 (PMC6226184; doi:10.1371/journal.pone.0207253)

Solanum species	Clone	Site	Date	Treatment	Sample id	Gel id	Lane id	PRI 10kDa	PR2+3 10-30kDa
					ladder	1	1		
S. dulcamara		Alnarp pond	2010-06-09		d.1.2.0	1	2	0	3
S. dulcamara		Lund genetikum	2010-06-14		d.2.2.0	1	3	0	0
S. dulcamara		Alnarp pond	2010-06-16		d.1.15.0	1	4	0	0
S. dulcamara		Lomma 1	2010-06-17		d.3.11.0	1	5	0	0
S. dulcamara		Lomma 2	2010-06-21		d.4.5.0	1	6	0	1
sample not included in the current study						1	7		
S. tuberosum	Bintje	Alnarp exp garden	2010-06-18	untreated	p.2.2.B6.0	1	8	0	0
S. tuberosum	Ovatio	Alnarp expgarden	2010-06-18	untreated	p.2.1.O2	1	9	0	0
S. tuberosum	Superb	Alnarp expgarden	2010-06-18	untreated	p.2.1.S1	1	10	0	0
S. dulcamara		Alnarp pond	2010-06-16		d.1.15.0	1b	1	0	0
S. tuberosum	Superb	Alnarp exp garden	2010-06-18	untreated	p.2:1.S1.0	1b	2	0	0
S. dulcamara		Alnarp pond	2010-06-09		d.1.2.0	1b	3	0	1
sample not included in the current study						1b	4		
S. tuberosum	Bintje	Alnarp expgarden	2010-06-18	untreated	p.2:2.B6.0	1b	5	0	0
S. tuberosum	Ovatio	Alnarp expgarden	2010-06-18	untreated	p.2:1.O2.0	1b	6	0	0
S. dulcamara		Lund genetikum	2010-06-14		d.2.2.0	1b	7	0	0
S. dulcamara		Lomma 2	2010-06-21		d.4.5.0	1b	8	0	1
S. dulcamara		Lomma 1	2010-06-21		d.3.11.0	1b	9	0	0
					ladder	1b	10		
					ladder	2	1		
S. dulcamara		Lomma 2	2010-07-27		d.4.12.0	2	2	N/A	N/A
S. dulcamara		Lund genetikum	2010-07-27		d.2.18.0	2	3	0	0
S. tuberosum	Bintje	Alnarp exp garden	2010-07-15	untreated	p.2.B9.0	2	4	0	0
S. tuberosum	Ovatio	Alnarp exp garden	2010-07-15	untreated	p.2.O4.0	2	5	0	0
S. tuberosum	Bintje	Borgeby	2010-07-20	untreated	p.4.B3.0	2	6	0	0
S. tuberosum	Ovatio	Borgeby	2010-07-20	untreated	p.4.O6.0	2	7	0	1
S. tuberosum	Bintje	Borgeby	2010-07-21	untreated	p.4.B11.0	2	8	0	0
positive control						2	9		

					ladder	2b	1		
S. dulcamara		Lund genetikum	2010-07-27		d.2.18.0	2b	2	0	0
S. tuberosum	Ovatio	Alnarp exp garden	2010-07-15	untreated	p.4.O6.0	2b	3	0	1
S. tuberosum	Ovatio	Alnarp exp garden	2010-07-15	untreated	p.2.O4.0	2b	4	0	0
S. tuberosum	Ovatio	Mosslunda	2010-07-14	untreated	p.3.O2.0	2b	5	0	0
S. dulcamara		Lomma 2	2010-07-27		d.4.12.0	2b	6	0	1
S. tuberosum	Bintje	Alnarp exp garden	2010-07-15	untreated	p.2.B9.0	2b	7	0	0
S. tuberosum	Bintje	Borgeby	2010-07-20	untreated	p.4.B3.0	2b	8	0	0
S. tuberosum	Bintje	Borgeby	2010-07-21	untreated	p.4.B11.0	2b	9	0	0
S. tuberosum	Bintje	Mosslunda	2010-07-14	untreated	p.3.B3.0	2b	10	0	0
					ladder	3	1		
S. dulcamara		Lomma 2	2010-08-27		d.4.13.0	3	2	0	1
S. nigrum		Alnarp south	2010-08-03		n.1.5.0	3	3	0	0
S. tuberosum	Binje	Alnarp exp garden	2010-08-09	untreated	p.2.B2.0	3	4	0	0
S. tuberosum	Ovatio	Alnarp exp garden	2010-08-09	untreated	p.2.O10.0	3	5	0	0
S. tuberosum	Binje	Borgeby	2010-08-17	untreated	p.4.B2.0	3	6	0	0
S. tuberosum	Ovatio	Borgeby	2010-08-17	untreated	p.4.O3.0	3	7	1	1
S. tuberosum	Sarpo Mira	Borgeby	2010-08-17	untreated	p.4.SM5.0	3	8	0	1
S. tuberosum	SW93-1015	Borgeby	2010-08-17	untreated	p.4.1015-16.0	3	9	0	0
positive control						3	10		
					ladder	3b	1		
S. nigrum		Alnarp south	2010-08-03		n.1.5.0	3b	2	0	0
S. tuberosum	Ovatio	Alnarp exp garden	2010-08-09	untreated	p.2.O10.0	3b	3	0	0
S. tuberosum	SW93-1015	Borgeby	2010-08-17	untreated	p.4.1015-16.0	3b	4	0	0
S. tuberosum	Ovatio	Borgeby	2010-08-17	untreated	p.4.O3.0	3b	5	1	1
S. tuberosum	Sarpo Mira	Borgeby	2010-08-17	untreated	p.4.SM5.0	3b	6	0	1
S. dulcamara		Lund genetikum	2010-08-27		d.2.24.0	3b	7	0	1
S. tuberosum	Binjte	Borgeby	2010-08-17	untreated	p.4.B2.0	3b	8	0	0
S. dulcamara		Lomma 2	2010-08-27		d.4.16.0	3b	9	0	0
S. tuberosum	Binjte	Alnarp exp garden	2010-08-09	untreated	p.2.B2.0	3b	10	0	0

			ladder	4	1			
S. dulcamara	Alnarp pond	2010-06-10	d.1.9.0	4	2	0	N/A
S. dulcamara	Lund genetikum	2010-06-14	d.2.11.0	4	3	N/A	N/A
S. dulcamara	Alnarp pond	2010-06-16	d.1.16.0	4	4	0	0
S. dulcamara	Lomma 1	2010-06-17	d.3.8.0	4	5	0	0
S. dulcamara	Lomma 2	2010-06-21	d.4.4.0	4	6	0	1
S. dulcamara	Lomma 2	2010-07-27	d.4.9.0	4	7	N/A	N/A
S. dulcamara	Lund genetikum	2010-07-27	d.2.13.0	4	8	0	1
S. dulcamara	Lomma 2	2010-08-27	d.4.18.0	4	9	0	0
positive control				4	10		
			ladder	4b	1		
S. dulcamara	Lomma 2	2010-08-27	d.4.18.0	4b	2	N/A	0
S. dulcamara	Lund genetikum	2010-08-27	d.2.21.0	4b	3	N/A	1
S. dulcamara	Lomma 2	2010-06-21	d.4.4.0	4b	4	N/A	0
S. dulcamara	Alnarp pond	2010-06-10	d.1.9.0	4b	5	N/A	0
S. dulcamara	Alnarp pond	2010-06-16	d.1.16.0	4b	6	N/A	0
S. dulcamara	Lund genetikum	2010-07-27	d.2.13.0	4b	7	N/A	1
S. dulcamara	Lomma 2	2010-07-27	d.4.9.0	4b	8	N/A	1
S. dulcamara	Lomma 1	2010-06-17	d.3.8.0	4b	9	N/A	0
S. dulcamara	Lund genetikum	2010-06-14	d.2.1.0	4b	10	N/A	1
			ladder	5	1		
S. dulcamara	Alnarp pond	2010-06-09	d.1.1.0	5	2	0	0
S. dulcamara	Lund genetikum	2010-06-14	d.2.6.0	5	3	0	1
S. dulcamara	Alnarp pond	2010-06-16	d.1.11.0	5	4	0	0
S. dulcamara	Lomma 1	2010-06-17	d.3.7.0	5	5	0	0
S. dulcamara	Lomma 2	2010-06-21	d.4.1.0	5	6	0	0
S. dulcamara	Lomma 2	2010-07-27	d.4.7.0	5	7	0	0
S. dulcamara	Lund genetikum	2010-07-27	d.2.14.0	5	8	0	0
S. dulcamara	Lomma 2	2010-08-27	d.4.17.0	5	9	0	1
S. dulcamara	Lund genetikum	2010-08-27	d.2.19.0	5	10	0	1

			ladder	6	1		
S. dulcamara	Alnarp pond	2010-06-10	d.1.7.0	6	2	0	0
S. dulcamara	Lund genetikum	2010-06-14	d.2.3.0	6	3	0	1
S. dulcamara	Lund genetikum	2010-06-14	d.2.5.0	6	4	0	0
S. dulcamara	Alnarp pond	2010-06-16	d.1.12.0	6	5	0	0
S. dulcamara	Lomma 1	2010-06-17	d.3.9.0	6	6	0	0
S. dulcamara	Lomma 2	2010-06-21	d.4.2.0	6	7	0	1
S. dulcamara	Lomma 2	2010-07-27	d.4.8.0	6	8	0	1
S. dulcamara	Lund genetikum	2010-07-27	d.2.15.0	6	9	0	1
S. dulcamara	Lund genetikum	2010-08-27	d.2.20.0	6	10	0	0
			ladder	7	1		
S. dulcamara	Alnarp pond	2010-06-09	d.1.5.0	7	2	0	0
S. dulcamara	Lund genetikum	2010-06-14	d.2.9.0	7	3	0	1
S. dulcamara	Lund genetikum	2010-06-14	d.2.10.0	7	4	0	0
S. dulcamara	Alnarp pond	2010-06-16	d.1.13.0	7	5	0	0
S. dulcamara	Lomma 1	2010-06-17	d.3.10.0	7	6	0	0
S. dulcamara	Lomma 2	2010-06-21	d.4.3.0	7	7	0	0
S. dulcamara	Lomma 2	2010-07-27	d.4.10.0	7	8	0	1
S. dulcamara	Lund genetikum	2010-07-27	d.2.16.0	7	9	0	1
S. dulcamara	Lund genetikum	2010-08-27	d.2.23.0	7	10	0	1
			ladder	8	1		
S. dulcamara	Alnarp pond	2010-06-09	d.1.3.0	8	2	0	0
S. dulcamara	Alnarp pond	2010-06-09	d.1.4.0	8	3	0	0
S. dulcamara	Lund genetikum	2010-06-14	d.2.4.0	8	4	0	1
S. dulcamara	Lund genetikum	2010-06-14	d.2.12.0	8	5	0	0
S. dulcamara	Alnarp pond	2010-06-16	d.1.14.0	8	6	0	0
S. dulcamara	Lomma 1	2010-06-17	d.3.12.0	8	7	0	0
S. dulcamara	Lomma 2	2010-06-21	d.4.6.0	8	8	0	0
S. dulcamara	Lomma 2	2010-07-27	d.4.11.0	8	9	0	1
S. dulcamara	Lund genetikum	2010-07-27	d.2.17.0	8	10	0	1

					ladder	9	1		
S. tuberosum	Bintje	Alnarp exp garden	2010-06-18	untreated	p.2:1.B1.0	9	2	0	0
S. tuberosum	Ovatio	Alnarp exp garden	2010-06-18	untreated	p.2:1.O1.0	9	3	0	1
S. tuberosum	Superb	Alnarp exp garden	2010-06-18	untreated	p.2:1.S2.0	9	4	0	0
S. tuberosum	Bintje	Alnarp exp garden	2010-06-18	untreated	p.2:2.B4.0	9	5	0	0
S. tuberosum	Bintje	Alnarp exp garden	2010-07-15	untreated	p.2.B1.0	9	6	0	0
S. tuberosum	Ovatio	Alnarp exp garden	2010-07-15	untreated	p.2.O5.0	9	7	0	1
S. tuberosum	Bintje	Alnarp exp garden	2010-08-09	untreated	p.2.B1.0	9	8	0	0
S. tuberosum	Ovatio	Alnarp exp garden	2010-08-09		p.2.O4.0	9	9	1	1
sample not included in the current study						9	10		
					ladder	10	1		
S. tuberosum	Ovatio	Alnarp exp garden	2010-06-18	untreated	p.2:1.O3.0	10	2	1	1
S. tuberosum	Bintje	Alnarp exp garden	2010-06-18	untreated	p.2:2.B5.0	10	3	0	0
S. tuberosum	Ovatio	Alnarp exp garden	2010-06-18	untreated	p.2:2.O6.0	10	4	0	1
S. tuberosum	Superb	Alnarp exp garden	2010-06-18	untreated	p.2:2.S4.0	10	5	0	0
S. tuberosum	Bintje	Alnarp exp garden	2010-07-15	untreated	p.2.B2.0	10	6	0	0
S. tuberosum	Ovatio	Alnarp exp garden	2010-07-15	untreated	p.2.O6.0	10	7	0	0
S. tuberosum	Bintje	Alnarp exp garden	2010-08-09	untreated	p.2.B3.0	10	8	0	0
S. tuberosum	Ovatio	Alnarp exp garden	2010-08-09		p.2.O5.0	10	9	1	1
sample not included in the current study						10	10		
					ladder	11	1		
S. tuberosum	Bintje	Alnarp exp garden	2010-06-18	untreated	p.2:1.B2.0	11	2	0	0
S. tuberosum	Superb	Alnarp exp garden	2010-06-18	untreated	p.2:1.S3.0	11	3	0	0
S. tuberosum	Ovatio	Alnarp exp garden	2010-06-18	untreated	p.2:2.O5.0	11	4	1	1
S. tuberosum	Superb	Alnarp exp garden	2010-06-18	untreated	p.2:2.S5.0	11	5	0	0
S. tuberosum	Bintje	Alnarp exp garden	2010-07-15	untreated	p.2.B3.0	11	6	0	0
S. tuberosum	Ovatio	Alnarp exp garden	2010-07-15	untreated	p.2.O10.0	11	7	0	0
S. tuberosum	Bintje	Alnarp exp garden	2010-08-09	untreated	p.2.B7.0	11	8	0	0
S. tuberosum	Ovatio	Alnarp exp garden	2010-08-09	untreated	p.2.O6.0	11	9	1	1
sample not included in the current study						11	10		

					ladder	12	1		
S. tuberosum	Bintje	Alnarp exp garden	2010-06-18	untreated	p.2:1.B3.0	12	2	0	0
S. tuberosum	Ovatio	Alnarp exp garden	2010-06-18	untreated	p.2:2.O4.0	12	3	0	0
S. tuberosum	Superb	Alnarp exp garden	2010-06-18	untreated	p.2:2.S6.0	12	4	0	0
S. tuberosum	Bintje	Alnarp exp garden	2010-07-15	untreated	p.2.B7.0	12	5	0	0
S. tuberosum	Ovatio	Alnarp exp garden	2010-07-15	untreated	p.2.O11.0	12	6	0	1
S. tuberosum	Bintje	Alnarp exp garden	2010-08-09	untreated	p.2.B8.0	12	7	0	0
S. tuberosum	Ovatio	Alnarp exp garden	2010-08-09	untreated	p.2.O11.0	12	8	1	1
sample not included in the current study						12	9		
sample not included in the current study						12	10		
					ladder	13	1		
S. tuberosum	Bintje	Borgeby	2010-08-17	untreated	p.4.B1.0	13	2	1	0
S. tuberosum	Ovatio	Borgeby	2010-08-17	untreated	p.4.O4.0	13	3	1	1
S. tuberosum	Ovatio	Borgeby	2010-07-21	untreated	p.4.O7.0	13	4	1	1
S. tuberosum	Sarpo Mira	Borgeby	2010-07-21	untreated	p.4.SM4.0	13	5	1	1
S. tuberosum	SW93-1015	Borgeby	2010-07-21	untreated	p.4.1015-10.0	13	6	0	0
S. tuberosum	Bintje	Borgeby	2010-08-17	untreated	p.4.B10.0	13	7	0	1
S. tuberosum	Ovatio	Borgeby	2010-08-17	untreated	p.4.O12.0	13	8	0	0
S. tuberosum	Sarpo Mira	Borgeby	2010-08-17	untreated	p.4.SM14.0	13	9	1	1
S. tuberosum	SW93-1015	Borgeby	2010-08-17	untreated	p.4.1015-8.0	13	10	0	0
					ladder	14	1		
S. tuberosum	Bintje	Borgeby	2010-07-20	untreated	p.4.B2.0	14	2	0	0
S. tuberosum	Ovatio	Borgeby	2010-07-21	untreated	p.4.O8.0	14	3	0	0
S. tuberosum	Sarpo Mira	Borgeby	2010-07-21	untreated	p.4.SM5.0	14	4	0	0
S. tuberosum	SW93-1015	Borgeby	2010-07-20	untreated	p.4.1015-11.0	14	5	0	N/A
S. tuberosum	SW93-1015	Borgeby	2010-07-21	untreated	p.4.1015-1.0	14	6	0	0
S. tuberosum	Bintje	Borgeby	2010-08-17	untreated	p.4.B1.0	14	7	1	0
S. tuberosum	Ovatio	Borgeby	2010-08-17	untreated	p.4.O4.0	14	8	1	1
S. tuberosum	Sarpo Mira	Borgeby	2010-08-17	untreated	p.4.SM6.0	14	9	0	1
S. tuberosum	SW93-1015	Borgeby	2010-08-17	untreated	p.4.1015-7.0	14	10	1	0

					ladder	15	1		
S. tuberosum	Bintje	Borgeby	2010-07-20	untreated	p.4.B2.0	15	2	0	0
S. tuberosum	Ovatio	Borgeby	2010-07-20	untreated	p.4.O5.0	15	3	0	1
S. tuberosum	Sarpo Mira	Borgeby	2010-07-20	untreated	p.4.SM9.0	15	4	0	1
S. tuberosum	SW93-1015	Borgeby	2010-07-20	untreated	p.4.1015-12.0	15	5	1	1
S. tuberosum	Bintje	Borgeby	2010-07-21	untreated	p.4.B12.0	15	6	0	0
S. tuberosum	Ovatio	Borgeby	2010-07-21	untreated	p.4.O9.0	15	7	0	1
S. tuberosum	Sarpo Mira	Borgeby	2010-07-21	untreated	p.4.SM6.0	15	8	0	1
S. tuberosum	SW93-1015	Borgeby	2010-07-21	untreated	p.4.1015-3.0	15	9	0	0
positive control						15	10		
					ladder	16	1		
sample not included in the current study						16	2		
sample not included in the current study						16	3		
sample not included in the current study						16	4		
sample not included in the current study						16	5		
sample not included in the current study						16	6		
sample not included in the current study						16	7		
S. tuberosum	Bintje	Mosslunda	2010-07-14	untreated	p.3.B1.0	16	8	0	0
S. tuberosum	Ovatio	Mosslunda	2010-07-14	untreated	p.3.O4.0	16	9	0	1
positive control						16	10		
					ladder	17	1		
S. tuberosum	Bintje	Mosslunda	2010-07-14	untreated	p.3.B5.0	17	2	0	0
S. tuberosum	Ovatio	Mosslunda	2010-07-14	untreated	p.3.O6.0	17	3	0	1
S. tuberosum	Bintje	Alnarp exp garden	2010-07-15	untreated	p.2.B8.0	17	4	0	0
S. tuberosum	Ovatio	Alnarp exp garden	2010-07-15	untreated	p.2.O12.0	17	5	0	0
S. tuberosum	Bintje	Borgeby	2010-07-20	untreated	p.4.B1.0	17	6	0	0
S. tuberosum	Ovatio	Borgeby	2010-07-20	untreated	p.4.O4.0	17	7	0	1
positive control						17	8		
					ladder	18	1		
S. dulcamara		Alnarp pond	2010-06-10		d.1.10.0	18	2	0	0

S. dulcamara		Lund genetikum	2010-06-14		d.2.8.0	18	3	0	0
S. nigrum		Alnarp south	2010-08-03		n.1.1.0	18	4	1	0
S. nigrum		Alnarp south	2010-08-03		n.1.2.0	18	5	0	0
S. nigrum		Alnarp south	2010-08-03		n.1.3.0	18	6	1	0
S. nigrum		Alnarp south	2010-08-03		n.1.4.0	18	7	1	0
S. nigrum		Alnarp south	2010-08-03		n.1.6.0	18	8	1	1
S. tuberosum	Bintje	Alnarp exp garden	2010-08-09	untreated	p.2.B9.0	18	9	0	0
S. tuberosum	Ovatio	Alnarp exp garden	2010-08-09	untreated	p.2.O12.0	18	10	1	1

Gel id

Code

0 = absence

1 = presence

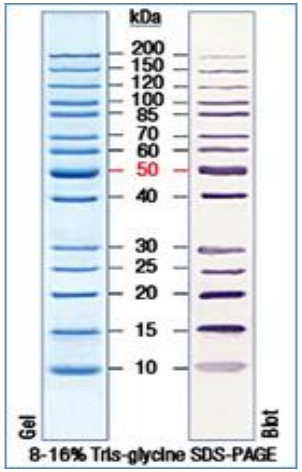

1

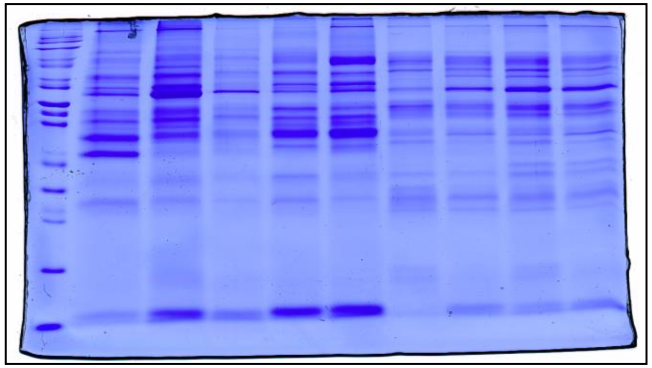

1b

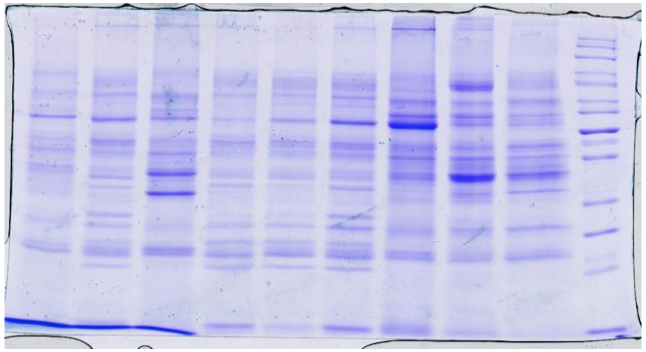

N/A = uncertain due to poor quality/presence of rubisco

2

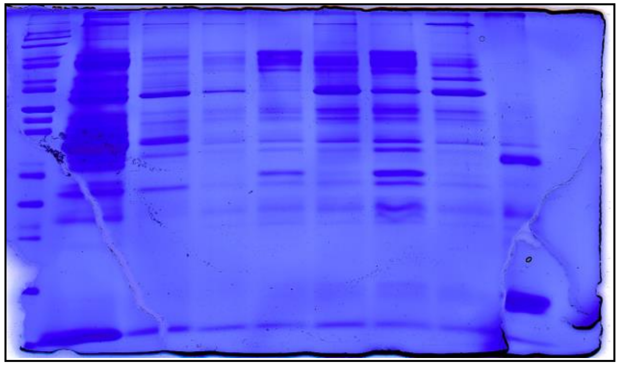

2b

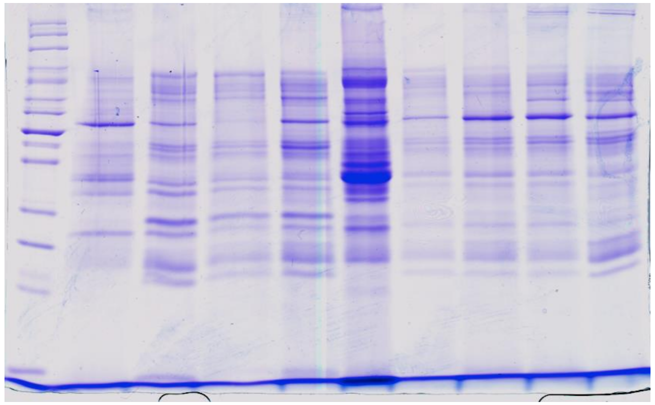

3

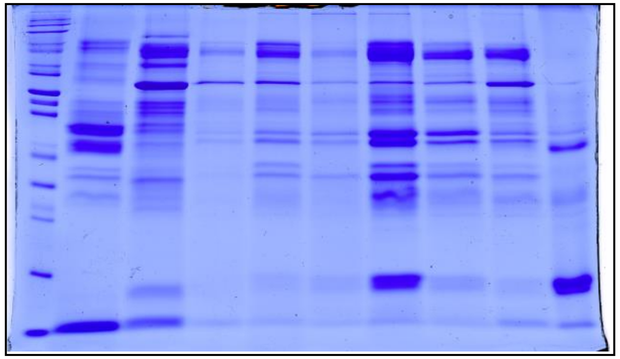

3b

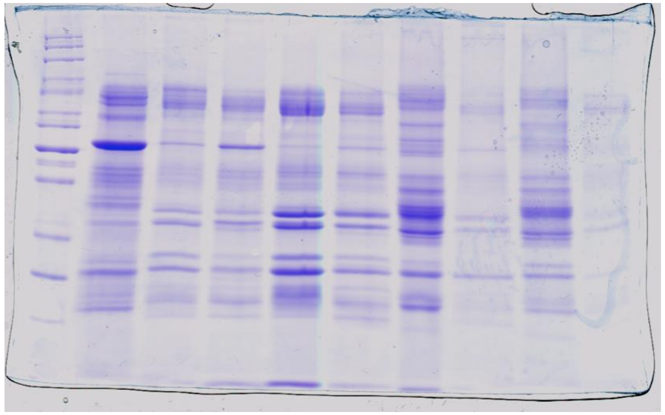

4

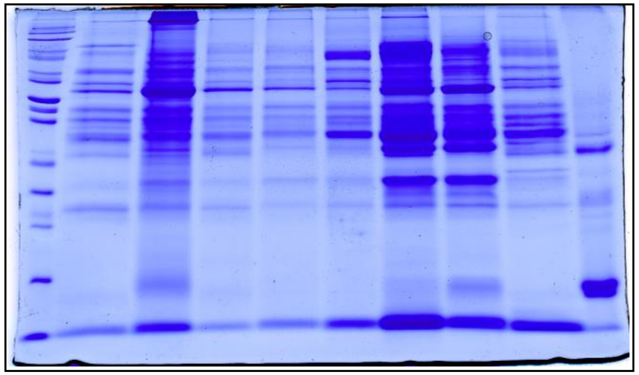

4b

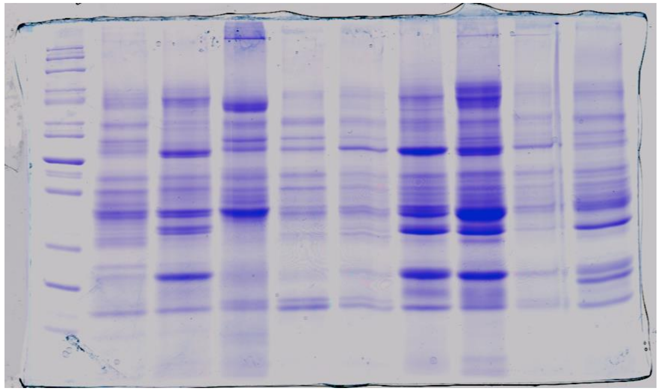

5

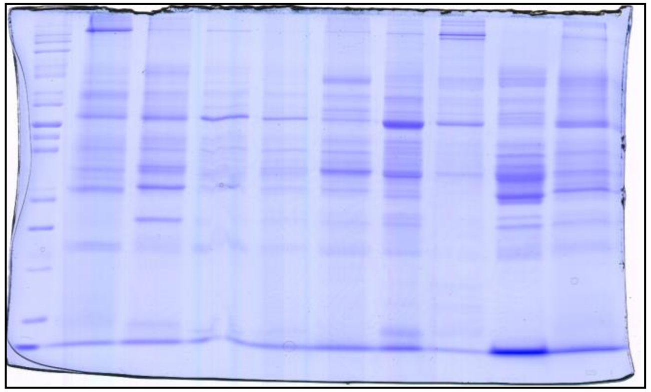

6

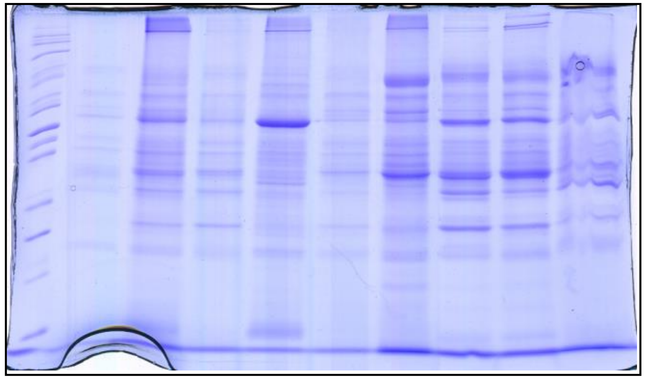

7

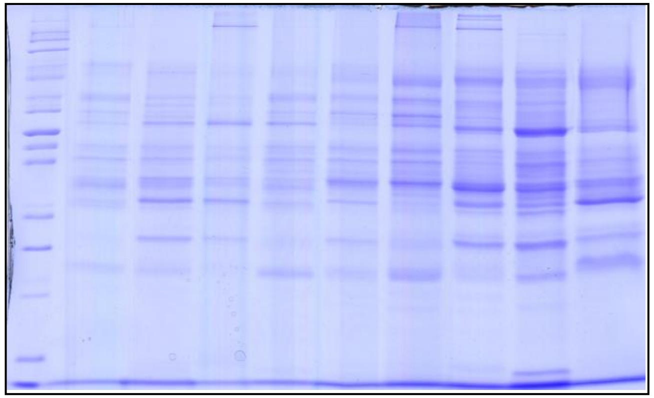

8

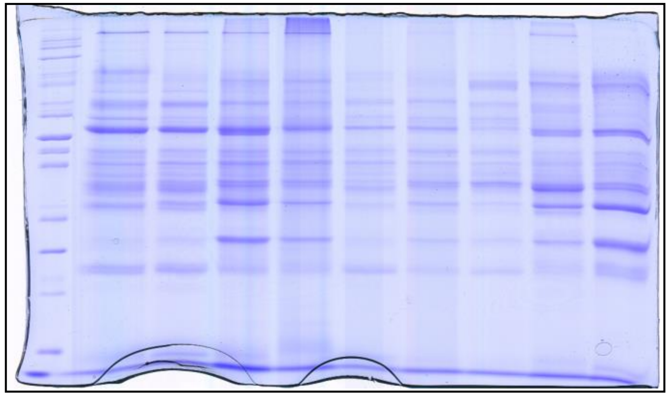

9

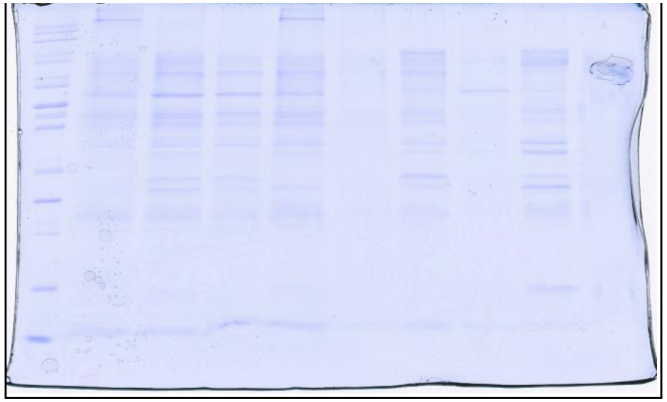

10

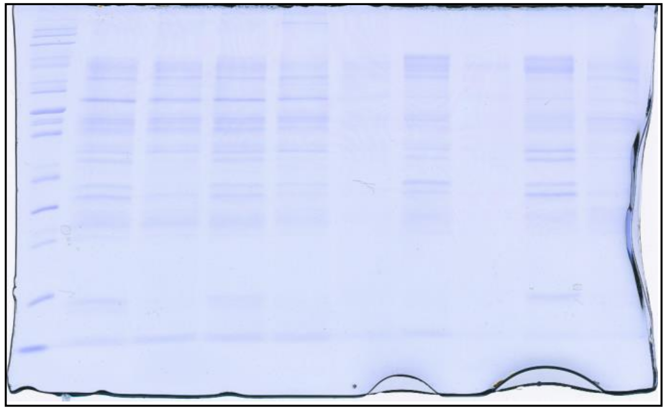

11

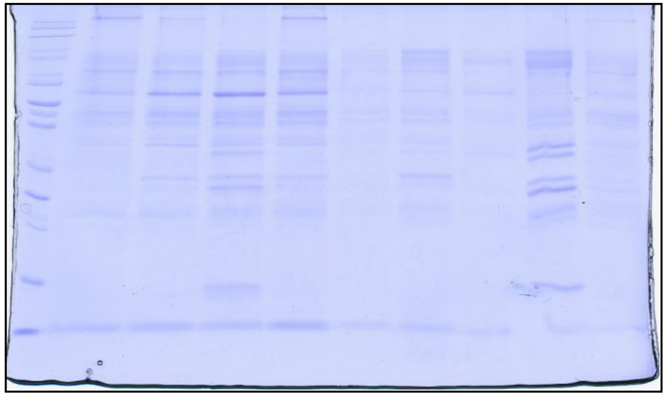

12

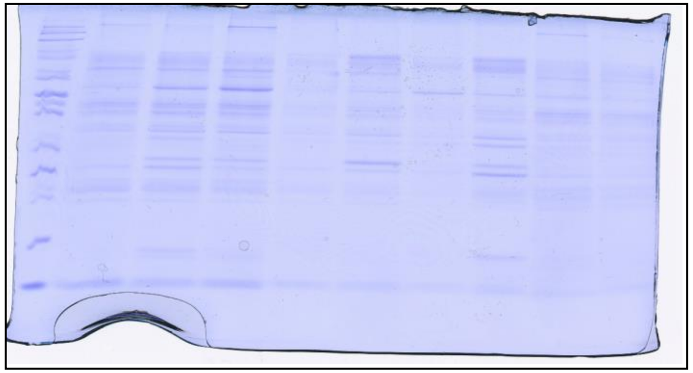

13

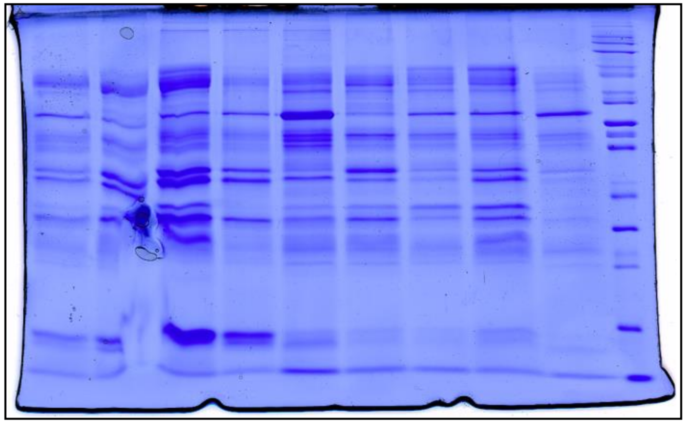

14

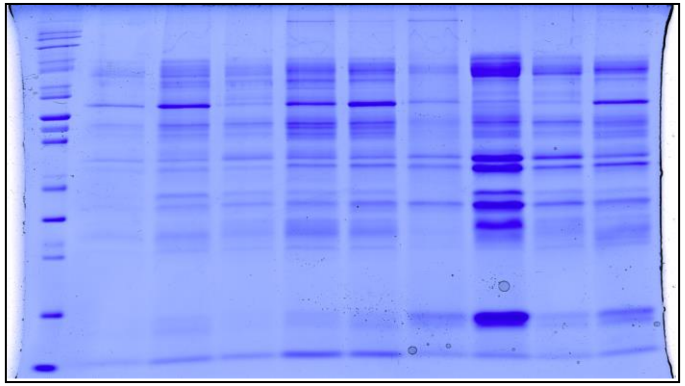

15

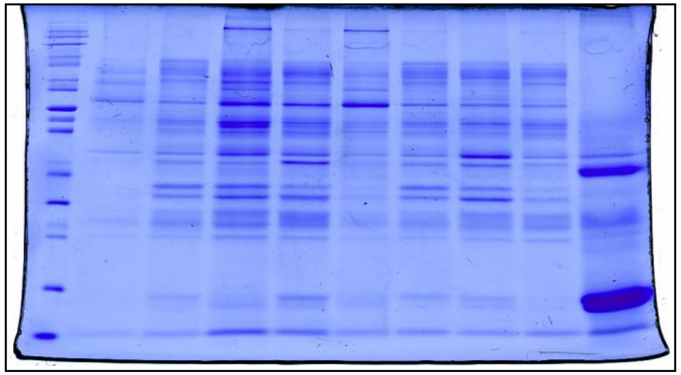

16

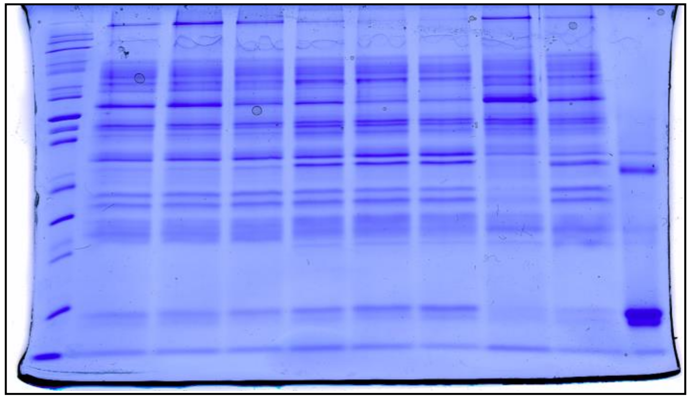

17

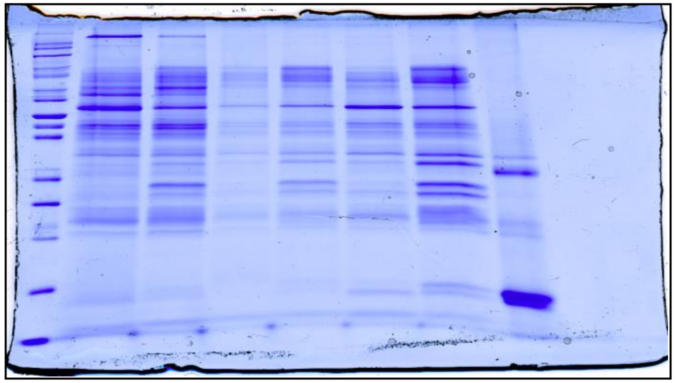

18

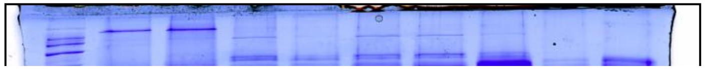

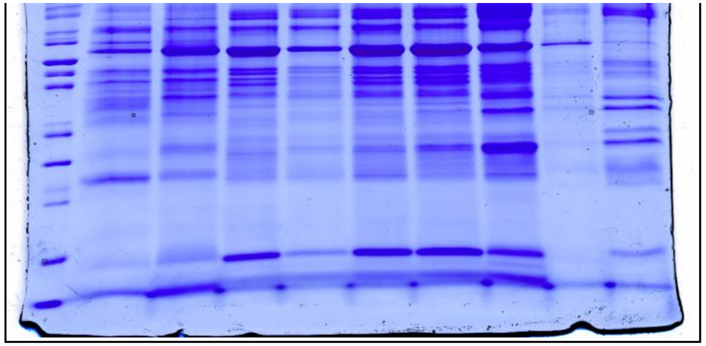

Supplement: S1 Dataset — (PDF) [file pone.0207253.s003.pdf]
