# Supplementary material for: Plant immunity in natural populations and agricultural fields: Low presence of pathogenesis-related proteins in Solanum leaves
Source: PLoS One. 2018 Nov 9;13(11):e0207253. doi: 10.1371/journal.pone.0207253 (PMC6226184; doi:10.1371/journal.pone.0207253)

Solanum species	Clone	Site	Date	Treatment	Sample id	Gel id	Lane id	PRI 10kDa	PR2+3 10-30kDa
					ladder	1	1		
S. dulcamara		Lund genetikum	2011-07-19		d322	1	2	0	1
S. dulcamara		Lund genetikum	2011-07-19		d327	1	3	0	1
S. dulcamara		Lund genetikum	2011-07-19		d330	1	4	0	0
S. dulcamara		Lund genetikum	2011-07-19		d334	1	5	0	0
S. dulcamara		Lund genetikum	2011-07-19		d339	1	6	0	1
S. dulcamara		Lund genetikum	2011-07-19		d343	1	7	0	0
					ladder	1b	1		
S. tuberosum	Ovatio	Mosslunda	2011-06-14	untreated	pO1	1b	2	0	0
S. tuberosum	Ovatio	Mosslunda	2011-06-14	untreated	pO2	1b	3	0	0
S. tuberosum	Ovatio	Mosslunda	2011-06-14	untreated	pO3	1b	4	0	0
S. tuberosum	Ovatio	Mosslunda	2011-06-14	phosphyte	pO4	1b	5	0	0
S. tuberosum	Ovatio	Mosslunda	2011-06-14	phosphyte	pO5	1b	6	0	0
S. tuberosum	Ovatio	Mosslunda	2011-06-14	phosphyte	pO6	1b	7	0	0
S. tuberosum	Ovatio	Mosslunda	2011-06-14	β-aminobutyric acid	pO7	1b	8	0	0
S. tuberosum	Ovatio	Mosslunda	2011-06-14	β-aminobutyric acid	pO8	1b	9	0	0
S. tuberosum	Bintje	Mosslunda	2011-06-14	untreated	pB1	1b	10	0	0
					ladder	2	1		
S. dulcamara		Lomma 1	2011-07-19		d299	2	2	0	1
S. dulcamara		Lomma 1	2011-07-19		d302	2	3	1	1
S. dulcamara		Lomma 1	2011-07-19		d306	2	4	0	1
S. dulcamara		Lomma 1	2011-07-19		d310	2	5	0	0
S. dulcamara		Lomma 1	2011-07-19		d314	2	6	0	0
S. dulcamara		Lomma 1	2011-07-19		d318	2	7	1	1

					ladder	2b	1		
S. tuberosum	Bintje	Mosslunda	2011-06-14	untreated	pB3	2b	2	0	0
S. tuberosum	Bintje	Mosslunda	2011-06-14	phosphyte	pB4	2b	3	0	0
S. tuberosum	Bintje	Mosslunda	2011-06-14	phosphyte	pB5	2b	4	0	0
S. tuberosum	Bintje	Mosslunda	2011-06-14	phosphyte	pB6	2b	5	0	0
S. tuberosum	Bintje	Mosslunda	2011-06-14	β-aminobutyric acid	pB7	2b	6	0	0
S. tuberosum	Bintje	Mosslunda	2011-06-14	β-aminobutyric acid	pB8	2b	7	0	0
S. tuberosum	Bintje	Mosslunda	2011-06-14	β-aminobutyric acid	pB9	2b	8	0	0
S. dulcamara		Lomma 3	2011-06-20		d78	2b	9	0	0
positive control						2b	10	1	1
					ladder	3	1		
					x	3	2		
S. dulcamara		Lomma 2	2011-07-20		d348	3	3	0	0
S. dulcamara		Lomma 2	2011-07-20		d351	3	4	0	1
S. dulcamara		Lomma 2	2011-07-20		d354	3	5	0	0
S. dulcamara		Lomma 2	2011-07-20		d359	3	6	1	1
S. dulcamara		Lomma 2	2011-07-20		d362	3	7	0	0
					ladder	3b	1		
S. dulcamara		Alnarp exp garden	2011-07-05		d211	3b	2	0	0
S. dulcamara		Alnarp exp garden	2011-07-05		d214	3b	3	0	0
S. dulcamara		Alnarp exp garden	2011-07-05		d221	3b	4	0	0
S. dulcamara		Alnarp exp garden	2011-07-05		d222	3b	5	0	0
S. dulcamara		Alnarp exp garden	2011-07-05		d234	3b	6	0	1
S. dulcamara		Alnarp exp garden	2011-07-05		d236	3b	7	0	1
S. dulcamara		Alnarp exp garden	2011-07-05		d238	3b	8	0	0
S. tuberosum	Ovatio	Mosslunda	2011-07-12	phosphyte	p57	3b	9	2	1
S. tuberosum	Desiree	Alnarp exp garden	2011-07-13	untreated	p75	3b	10	1	0

					ladder	4	1		
S. dulcamara		Lomma 3	2011-07-20		d371	4	2	0	0
S. dulcamara		Lomma 3	2011-07-20		d375	4	3	0	0
S. dulcamara		Lomma 3	2011-07-20		d378	4	4	0	0
					x	4	5		
					x	4	6		
					x	4	7		
S. dulcamara		Lomma 3	2011-07-20		d383	4	8	0	0
S. dulcamara		Lomma 3	2011-07-20		d387	4	9	1	1
S. dulcamara		Lomma 3	2011-07-20		d391	4	10	1	1
					ladder	4b	1		
S. tuberosum	SW93-1015	Alnarp exp garden	2011-07-13	untreated	p102	4b	2	0	0
S. tuberosum	Sarpo Mira	Alnarp exp garden	2011-07-13	untreated	p95	4b	3	0	0
S. tuberosum	Desiree	Alnarp exp garden	2011-07-13	untreated	p83	4b	4	0	0
S. tuberosum	SW93-1015	Alnarp exp garden	2011-07-13	untreated	p87	4b	5	0	0
S. tuberosum	Sarpo Mira	Alnarp exp garden	2011-07-13	untreated	p91	4b	6	0	0
S. tuberosum	Desiree	Alnarp exp garden	2011-07-13	untreated	p98	4b	7	0	0
S. tuberosum	SW93-1015	Alnarp exp garden	2011-07-13	untreated	p101	4b	8	0	0
S. tuberosum	Sarpo Mira	Alnarp exp garden	2011-07-13	untreated	p106	4b	9	0	0
S. nigrum		Alnarp exp garden	2011-07-13		n17	4b	10	N/A	N/A
					ladder	5	1		
S. dulcamara		Alnarp pond	2011-06-17		d1	5	2	0	0
S. dulcamara		Alnarp pond	2011-06-17		d6	5	3	0	0
					x	5	4		

S. dulcamara	Alnarp pond	2011-06-16	d14	5	5	0	1
S. dulcamara	Alnarp pond	2011-06-17	d17	5	6	0	0
S. dulcamara	Alnarp pond	2011-06-17	d38	5	7	1	1
			ladder	5b	1		
sample not included in the current study				5b	2		
sample not included in the current study				5b	3		
sample not included in the current study				5b	4		
sample not included in the current study				5b	5		
sample not included in the current study				5b	6		
sample not included in the current study				5b	7		
sample not included in the current study				5b	8		
sample not included in the current study				5b	9		
S. dulcamara	Lomma 3	2011-07-20	d370	5b	10	0	0
			ladder	6	1		
S. nigrum	Spillepengen	2011-06-14	n23	6	2	0	0
S. nigrum	Spillepengen	2011-06-14	n26	6	3	0	0
S. nigrum	Spillepengen	2011-06-14	n29	6	4	0	0
S. nigrum	Spillepengen	2011-06-14	n33	6	5	0	0
S. nigrum	Spillepengen	2011-06-14	n37	6	6	0	0
S. nigrum	Spillepengen	2011-06-14	n41	6	7	0	0
			ladder	6b	1		
S. dulcamara	Lomma 3	2011-07-20	d382	6b	2	0	0

sample not included in the current study				6b	3		
sample not included in the current study				6b	4		
			ladder	7	1		
S. dulcamara	Spillepengen	2011-06-14	d274	7	2	0	1
S. dulcamara	Spillepengen	2011-06-14	d279	7	3	0	0
S. dulcamara	Spillepengen	2011-06-14	d283	7	4	0	0
S. dulcamara	Spillepengen	2011-06-14	d286	7	5	0	0
S. dulcamara	Spillepengen	2011-06-14	d290	7	6	0	0
S. dulcamara	Spillepengen	2011-06-14	d295	7	7	0	0
			ladder	8	1		
S. nigrum	Alnarp exp garden	2011-07-05	n4	8	2	0	0
S. nigrum	Alnarp exp garden	2011-07-05	n6	8	3	0	0
S. nigrum	Alnarp exp garden	2011-07-05	n10	8	4	0	0
S. nigrum	Alnarp exp garden	2011-07-05	n13	8	5	0	0
S. dulcamara	Alnarp exp garden	2011-07-05	d227	8	6	1	1
S. dulcamara	Alnarp exp garden	2011-07-05	d246	8	7	0	0
			ladder	9	1		
S. dulcamara	Lomma 1	2011-06-21	d87	9	2	0	0
S. dulcamara	Lomma 1	2011-06-21	d95	9	3	0	0
S. dulcamara	Lomma 1	2011-06-21	d91	9	4	0	0
S. dulcamara	Lomma 1	2011-06-21	d99	9	5	0	0
S. dulcamara	Lomma 1	2011-06-21	d103	9	6	0	0
S. dulcamara	Lomma 1	2011-06-21	d108	9	7	1	1

S. dulcamara	Tvedöra	2011-06-21	d136	9	8	0	0
S. dulcamara	Tvedöra	2011-06-21	d140	9	9	0	0
S. dulcamara	Tvedöra	2011-06-21	d143	9	10	1	0
			ladder	10	1		
S. dulcamara	Tvedöra	2011-06-21	d149	10	2	0	0
S. dulcamara	Tvedöra	2011-06-21	d151	10	3	0	0
S. dulcamara	Tvedöra	2011-06-21	d158	10	4	0	0
S. dulcamara	Alnarp pond	2011-06-17	d4	10	5	N/A	N/A
S. nigrum	Alnarp exp garden	2011-06-16	n6:1	10	6	N/A	N/A
S. nigrum	Alnarp exp garden	2011-06-16	n10:1	10	7	0	0
S. dulcamara	Alnarp pond	2011-06-17	d13	10	8	0	1
S. dulcamara	Alnarp pond	2011-06-17	d18	10	9	0	0
S. dulcamara	Alnarp pond	2011-06-17	d22	10	10	0	0
			ladder	13	1		
S. dulcamara	Lomma 2	2011-06-20	d41	13	2	0	0
S. dulcamara	Lomma 2	2011-06-20	d57	13	3	0	0
S. dulcamara	Lomma 2	2011-06-20	d51	13	4	0	0
S. dulcamara	Lomma 2	2011-06-20	d52	13	5	0	0
S. dulcamara	Lomma 2	2011-06-20	d45	13	6	0	0
S. dulcamara	Lomma 2	2011-06-20	d60	13	7	0	0
S. dulcamara	Lund genetikum	2011-06-21	d134	13	8	0	0
S. dulcamara	Lund genetikum	2011-06-21	d115	13	9	0	0
S. dulcamara	Lund genetikum	2011-06-21	d119	13	10	0	0
			ladder	14	1		
S. dulcamara	Lund genetikum	2011-06-21	d122	14	2	0	0
S. dulcamara	Lund genetikum	2011-06-21	d126	14	3	0	0
S. dulcamara	Lund genetikum	2011-06-21	d132	14	4	N/A	N/A
S. dulcamara	Lomma 3	2011-06-20	d65	14	5	1	0

S. dulcamara		Lomma 3	2011-06-20		d69	14	6	1	0
S. dulcamara		Lomma 3	2011-06-20		d70	14	7	0	0
S. dulcamara		Lomma 3	2011-06-20		d73	14	8	0	0
S. dulcamara		Lomma 3	2011-06-20		d85	14	9	N/A	N/A
S. dulcamara		Lomma 3	2011-06-20		d82	14	10	0	1
					ladder	15	1		
S. dulcamara		Alnarp hedge	2011-06-22		d161	15	2	0	0
S. dulcamara		Alnarp hedge	2011-06-22		d166	15	3	0	0
S. dulcamara		Alnarp hedge	2011-06-22		d169	15	4	N/A	N/A
S. dulcamara		Alnarp hedge	2011-06-22		d173	15	5	0	0
S. dulcamara		Alnarp hedge	2011-06-22		d177	15	6	0	0
S. dulcamara		Alnarp hedge	2011-06-22		d182	15	7	0	0
					ladder	16	1		
S. dulcamara		Alnarp hedge	2011-07-04		d185	16	2	0	0
S. dulcamara		Alnarp hedge	2011-07-04		d191	16	3	0	1
S. dulcamara		Alnarp hedge	2011-07-04		d195	16	4	0	1
S. dulcamara		Alnarp hedge	2011-07-04		d198	16	5	0	1
S. dulcamara		Alnarp hedge	2011-07-04		d203	16	6	0	1
S. dulcamara		Alnarp hedge	2011-07-04		d206	16	7	N/A	N/A
					ladder	17	1		
S. tuberosum	Ovatio	Mosslunda	2011-07-12	untreated	p41	17	2	1	1
S. tuberosum	Bintje	Mosslunda	2011-07-12	β-aminobutyric acid	p30	17	3	0	0
S. tuberosum	Ovatio	Mosslunda	2011-07-12	phosphyte	p60	17	4	N/A	N/A
S. tuberosum	Ovatio	Mosslunda	2011-07-12	β-aminobutyric acid	p70	17	5	1	0
S. tuberosum	Bintje	Mosslunda	2011-07-12	phosphyte	p2	17	6	0	0

S. tuberosum	Ovatio	Mosslunda	2011-07-12	phosphyte	p53	17	7	0	0
S. tuberosum	Ovatio	Mosslunda	2011-07-12	β-aminobutyric acid	p62	17	8	0	0
S. tuberosum	Ovatio	Mosslunda	2011-07-12	phosphyte	p68	17	9	0	0
					ladder	18	1		
S. tuberosum	Ovatio	Mosslunda	2011-07-12	untreated	p25	18	2	0	1
S. tuberosum	Bintje	Mosslunda	2011-07-12	untreated	p6	18	3	N/A	N/A
S. tuberosum	Bintje	Mosslunda	2011-07-12	untreated	p10	18	4	0	1
S. tuberosum	Ovatio	Mosslunda	2011-07-12	untreated	p37	18	5	1	1
S. tuberosum	Ovatio	Mosslunda	2011-07-12	untreated	p41	18	6	1	1
S. tuberosum	Bintje	Mosslunda	2011-07-12	untreated	p22	18	7	0	0
					ladder	19	1		
S. dulcamara		Tvedöra	2011-07-20		d394	19	2	0	0
S. dulcamara		Tvedöra	2011-07-20		d398	19	3	0	0
S. dulcamara		Tvedöra	2011-07-20		d402	19	4	0	0
S. dulcamara		Tvedöra	2011-07-20		d406	19	5	0	1
S. dulcamara		Tvedöra	2011-07-20		d410	19	6	0	1
S. dulcamara		Tvedöra	2011-07-20		d414	19	7	0	1
					ladder	20	1		
S. dulcamara		Lomma 3	2011-07-20		d373	20	2	0	0
S. dulcamara		Lomma 3	2011-07-20		d377	20	3	0	0
S. dulcamara		Lomma 3	2011-08-17		d502	20	4	0	1
S. dulcamara		Lomma 3	2011-08-17		d507	20	5	0	1
S. dulcamara		Lomma 3	2011-08-17		d510	20	6	1	1
S. dulcamara		Lomma 3	2011-08-17		d514	20	7	0	0
S. dulcamara		Lomma 3	2011-08-17		d519	20	8	0	1
S. dulcamara		Lomma 3	2011-08-17		d522	20	9	0	0

S. tuberosum	Bintje	Mosslunda	2011-08-02	phosphyte	p181	20	10	1	0
					ladder	21	1		
S. dulcamara		Alnarp exp garden	2011-07-05		d210	21	2	0	0
S. dulcamara		Alnarp exp garden	2011-07-05		d215	21	**3**	0	0
S. dulcamara		Alnarp exp garden	2011-07-05		d218	21	4	0	0
S. dulcamara		Alnarp exp garden	2011-07-05		d223	21	5	0	0
S. dulcamara		Alnarp exp garden	2011-07-05		d242	21	6	0	0
S. dulcamara		Alnarp exp garden	2011-07-05		d230	21	**7**	0	0
S. dulcamara		Alnarp exp garden	2011-07-05		d235	21	8	0	1
S. dulcamara		Alnarp exp garden	2011-07-05		d239	21	9	0	0
					ladder	22	1		
sample not included in the current study						22	2		
sample not included in the current study						22	3		
sample not included in the current study						22	4		
sample not included in the current study						22	5		
S. nigrum		Alnarp exp garden	2011-07-05		n9	22	6	0	0
S. nigrum		Alnarp exp garden	2011-07-05		n14	22	7	0	0
S. dulcamara		Alnarp exp garden	2011-08-01		d420	22	8	1	1
sample not included in the current study							9		
					ladder	23	1		
S. tuberosum	Ovatio	Mosslunda	2011-06-14	untreated	pO1	23	2	0	0
S. tuberosum	Ovatio	Mosslunda	2011-06-14	untreated	pO2	23	3	0	0
S. tuberosum	Ovatio	Mosslunda	2011-06-14	untreated	pO3	23	4	0	0
S. tuberosum	Ovatio	Mosslunda	2011-06-14	phosphyte	pO4	23	5	0	0
S. tuberosum	Ovatio	Mosslunda	2011-06-14	phosphyte	pO5	23	6	0	0
S. tuberosum	Ovatio	Mosslunda	2011-06-14	phosphyte	pO6	23	7	0	0

S. tuberosum	Ovatio	Mosslunda	2011-06-14	β-aminobutyric acid	pO7	23	8	0	0
S. tuberosum	Ovatio	Mosslunda	2011-06-14	β-aminobutyric acid	pO8	23	9	0	0
S. tuberosum	Ovatio	Mosslunda	2011-06-14	β-aminobutyric acid	pO9	23	10	N/A	N/A
					ladder	24	1		
S. tuberosum	Bintje	Mosslunda	2011-06-14	untreated	pB1	24	2	0	0
S. tuberosum	Bintje	Mosslunda	2011-06-14	untreated	pB2	24	3	0	0
S. tuberosum	Ovatio	Mosslunda	2011-06-14	β-aminobutyric acid	p50	24	4	0	0
S. tuberosum	Bintje	Mosslunda	2011-06-14	untreated	pB3	24	5	0	0
S. tuberosum	Bintje	Mosslunda	2011-06-14	phosphyte	pB4	24	6	0	0
S. tuberosum	Bintje	Mosslunda	2011-06-14	phosphyte	pB5	24	7	0	0
S. tuberosum	Bintje	Mosslunda	2011-06-14	phosphyte	pB6	24	8	0	0
S. tuberosum	Bintje	Mosslunda	2011-06-14	β-aminobutyric acid	pB7	24	9	0	0
S. tuberosum	Bintje	Mosslunda	2011-06-14	β-aminobutyric acid	pB9	24	10	0	0
					ladder	25	1		
S. tuberosum	Bintje	Mosslunda	2011-06-14	β-aminobutyric acid	pB8	25	2	0	0
S. tuberosum	Bintje	Mosslunda	2011-07-17	β-aminobutyric acid	p34	25	3	0	0
S. tuberosum	Bintje	Mosslunda	2011-07-17	β-aminobutyric acid	p46	25	4	0	0
S. tuberosum	Bintje	Mosslunda	2011-07-17	untreated	p21	25	5	0	0
S. tuberosum	Bintje	Mosslunda	2011-07-17	untreated	p5	25	6	0	0
S. tuberosum	Bintje	Mosslunda	2011-07-17	untreated	p9	25	7	N/A	N/A
S. tuberosum	Bintje	Mosslunda	2011-07-17	β-aminobutyric acid	p162	25	8	1	0
S. tuberosum	Bintje	Mosslunda	2011-07-17	β-aminobutyric acid	p154	25	9	1	0
S. tuberosum	Bintje	Mosslunda	2011-07-17	β-aminobutyric acid	p146	25	10	1	0
					ladder	27	1		
S. dulcamara		Lomma 2	2011-08-08		d478	27	2	0	0
S. dulcamara		Lomma 2	2011-08-08		d482	27	3	0	1
S. dulcamara		Lomma 2	2011-08-08		d490	27	4	0	1
S. dulcamara		Lomma 2	2011-08-08		d494	27	5	0	0

S. dulcamara		Lomma 2	2011-08-08		d498	27	6	0	0
S. dulcamara		Alnarp hedge	2011-08-05		d357	27	7	0	1
S. dulcamara		Alnarp hedge	2011-08-05		d361	27	8	0	0
S. dulcamara		Alnarp hedge	2011-08-05		d365	27	9	0	0
S. dulcamara		Alnarp hedge	2011-08-05		d369	27	10	0	0
					ladder	28	1		
S. tuberosum	Bintje	Mosslunda	2011-08-02	phosphyte	p188	28	2	1	0
S. tuberosum	Bintje	Mosslunda	2011-08-02	phosphyte	p196	28	3	1	0
S. tuberosum	Bintje	Mosslunda	2011-08-02	β-aminobutyric acid	p201	28	4	1	0
S. tuberosum	Ovatio	Mosslunda	2011-08-02	β-aminobutyric acid	p204	28	5	1	1
S. tuberosum	Ovatio	Mosslunda	2011-08-02	phosphyte	p208	28	6	1	1
S. tuberosum	Ovatio	Mosslunda	2011-08-02	β-aminobutyric acid	p212	28	7	1	1
S. tuberosum	Ovatio	Mosslunda	2011-08-02	phosphyte	p218	28	8	1	1
S. tuberosum	Ovatio	Mosslunda	2011-08-02	phosphyte	p221	28	9	1	1
sample not included in the current study							10		
					ladder	29	1		
sample not included in the current study						29	2		
sample not included in the current study						29	3		
sample not included in the current study						29	4		
sample not included in the current study						29	5		
S. dulcamara		Alnarp pond	2011-08-18		d397	29	6	0	0
S. dulcamara		Alnarp pond	2011-08-18		d393	29	7	0	0
S. dulcamara		Alnarp pond	2011-08-18		d383	29	8	0	1
S. dulcamara		Alnarp pond	2011-08-18		d385	29	9	0	1
S. dulcamara		Alnarp pond	2011-08-18		d402	29	10	1	1
					ladder	30	1		
sample not included in the current study							2		
S. tuberosum	Bintje	Mosslunda	2011-07-12	phosphyte	p17	30	3	0	0
S. tuberosum	Bintje	Mosslunda	2011-07-12	phosphyte	p15	30	4	0	0

sample not included in the current study						30	5		
sample not included in the current study						30	6		
sample not included in the current study						30	7		
sample not included in the current study						30	8		
sample not included in the current study						30	9		
sample not included in the current study						30	10		
					ladder	31	1		
S. tuberosum	Sarpo Mira	Alnarp exp garden	2011-08-05	untreated	p352	31	2	0	1
S. tuberosum	Sarpo Mira	Alnarp exp garden	2011-08-05	untreated	p356	31	3	0	1
S. dulcamara		Lomma 1	2011-08-18		d405	31	4	0	0
S. dulcamara		Lomma 1	2011-08-18		d409	31	5	0	1
S. dulcamara		Lomma 1	2011-08-18		d413	31	6	0	0
S. dulcamara		Lomma 1	2011-08-18		d417	31	7	0	0
S. dulcamara		Lomma 1	2011-08-18		d421	31	8	0	0
S. dulcamara		Lomma 1	2011-08-18		d425	31	9	0	0
S. tuberosum	SW93-1015	Alnarp exp garden	2011-08-05	untreated	p324	31	10	0	0
					ladder	32	1		
S. tuberosum	SW93-1015	Alnarp exp garden	2011-08-05	untreated	p328	32	2	0	0
S. tuberosum	SW93-1015	Alnarp exp garden	2011-08-05	untreated	p332	32	3	0	0
S. tuberosum	Desiree	Alnarp exp garden	2011-08-05	untreated	p336	32	4	1	1
S. tuberosum	Desiree	Alnarp exp garden	2011-08-05	untreated	p340	32	5	1	1
S. tuberosum	Desiree	Alnarp exp garden	2011-08-05	untreated	p344	32	6	0	1
S. tuberosum	Sarpo Mira	Alnarp exp garden	2011-08-05	untreated	p348	32	7	0	0
S. dulcamara		Alnarp exp garden	2011-08-01		d418	32	8	1	1
S. dulcamara		Alnarp exp garden	2011-08-01		d422	32	9	0	0
S. dulcamara		Alnarp exp garden	2011-08-01		d426	32	10	0	0
					ladder	33	1		

S. dulcamara		Alnarp exp garden	2011-08-01		d428	33	2	0	0
S. dulcamara		Alnarp exp garden	2011-08-01		d432	33	3	0	1
S. dulcamara		Alnarp exp garden	2011-08-01		d435	33	4	0	0
S. dulcamara		Alnarp exp garden	2011-08-01		d437	33	5	0	0
S. dulcamara		Alnarp exp garden	2011-08-01		d441	33	6	0	0
S. tuberosum	Ovatio	Mosslunda	2011-08-02	β-aminobutyric acid	p225	33	7	1	1
S. tuberosum	SW93-1015	Borgeby	2011-08-04	untreated	p300	33	8	1	0
S. tuberosum	SW93-1015	Borgeby	2011-08-04	untreated	p305	33	9	1	0
S. tuberosum	SW93-1015	Borgeby	2011-08-04	untreated	p308	33	10	1	0
					ladder	34	1		
S. tuberosum	Sarpo Mira	Borgeby	2011-08-04	untreated	p313	34	2	1	1
S. tuberosum	Sarpo Mira	Borgeby	2011-08-04	untreated	p317	34	3	1	1
S. tuberosum	Sarpo Mira	Borgeby	2011-08-04	untreated	p321	34	4	1	1
S. nigrum		Borgeby	2011-08-04		n61	34	5	0	0
S. nigrum		Borgeby	2011-08-04		n64	34	6	0	0
S. nigrum		Borgeby	2011-08-04		n66	34	7	0	0
S. nigrum		Borgeby	2011-08-04		n70	34	8	0	0
S. nigrum		Borgeby	2011-08-04		n73	34	9	0	0
S. nigrum		Borgeby	2011-08-04		n76	34	10	0	0
					ladder	35	1		
S. dulcamara		Spillepengen	2011-08-11		d526	35	2	0	1
S. dulcamara		Spillepengen	2011-08-11		d529	35	3	0	1
S. dulcamara		Spillepengen	2011-08-11		d532	35	4	0	0
S. dulcamara		Spillepengen	2011-08-11		d533	35	5	0	0
S. dulcamara		Spillepengen	2011-08-11		d538	35	6	0	1
S. dulcamara		Spillepengen	2011-08-11		d541	35	7	0	1
S. nigrum		Spillepengen	2011-08-11		n102	35	8	0	0
S. nigrum		Spillepengen	2011-08-11		n105	35	9	0	0
S. nigrum		Spillepengen	2011-08-11		n108	35	10	0	0

			ladder	36	1		
S. nigrum	Alnarp exp garden	2011-08-01	n45	36	2	0	0
S. nigrum	Alnarp exp garden	2011-08-01	n48	36	3	1	0
S. nigrum	Alnarp exp garden	2011-08-01	n52	36	4	0	0
S. nigrum	Alnarp exp garden	2011-08-01	n57	36	5	0	0
S. dulcamara	Alnarp exp garden	2011-08-01	d250	36	6	N/A	N/A
S. dulcamara	Alnarp exp garden	2011-08-01	d254	36	7	1	0
S. dulcamara	Lund genetikum	2011-08-17	d429	36	8	0	0
S. dulcamara	Lund genetikum	2011-08-17	d433	36	9	N/A	N/A
S. dulcamara	Lund genetikum	2011-08-17	d441	36	10	N/A	N/A
			ladder	37	1		
S. nigrum	Spillepengen	2011-08-11	n111	37	2	0	0
S. nigrum	Spillepengen	2011-08-11	n114	37	3	1	1
S. nigrum	Spillepengen	2011-08-11	n117	37	4	0	0
S. nigrum	Alnarp south	2011-08-11	n78	37	5	0	0
S. nigrum	Alnarp south	2011-08-11	n84	37	6	0	0
S. nigrum	Alnarp south	2011-08-11	n81	37	7	0	0
S. nigrum	Alnarp south	2011-08-11	n87	37	8	0	1
S. nigrum	Alnarp south	2011-08-11	n91	37	9	0	1
S. nigrum	Alnarp south	2011-08-11	n93	37	10	0	1
			ladder	38	1		
S.dulcamara	Lund genetikum	2011-08-17	d445	38	2	0	1
S.dulcamara	Lund genetikum	2011-08-17	d449	38	3	0	1
S.dulcamara	Tvedöra	2011-08-17	d453	38	4	0	0
S.dulcamara	Tvedöra	2011-08-17	d457	38	5	0	1
S.dulcamara	Tvedöra	2011-08-17	d461	38	6	0	1
S.dulcamara	Tvedöra	2011-08-17	d465	38	7	0	0
S.dulcamara	Tvedöra	2011-08-17	d470	38	8	0	0

S.dulcamara	Tvedöra	2011-08-17	d474	38	9	0	0
S.dulcamara	Lomma 2	2011-08-08	d486	38	10	0	0

Gel id

1
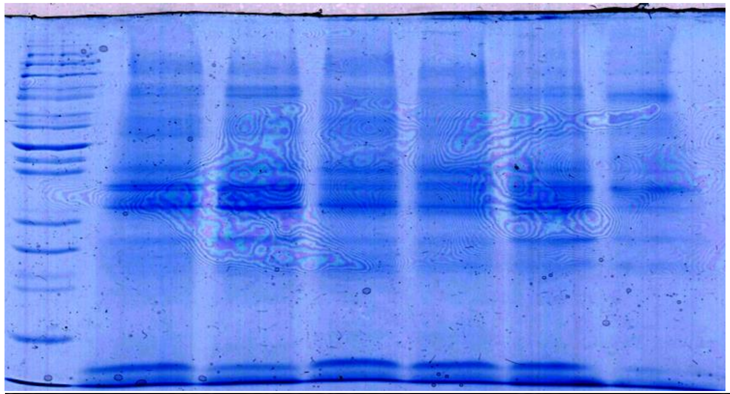

1b
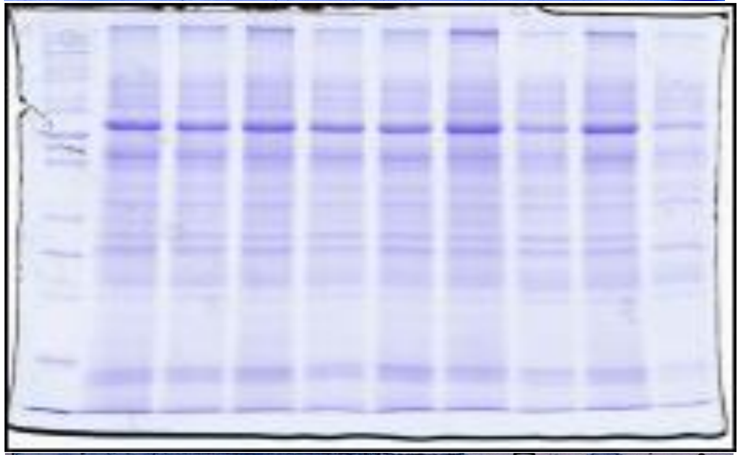

2
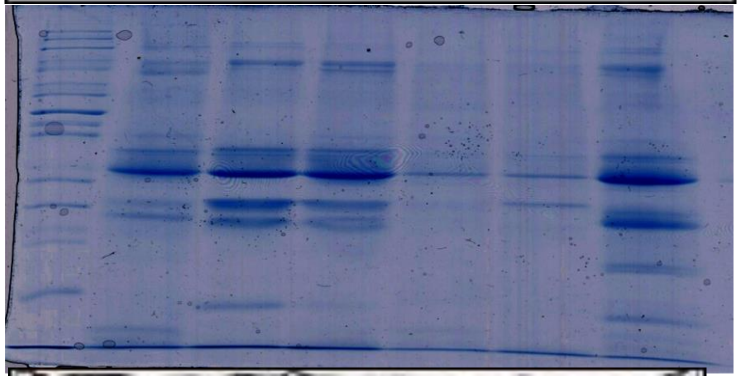

Code
0 = absence
1 = presence

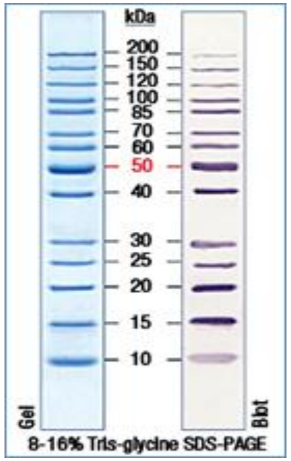

N/A = uncertain due to poor quality/presence of rubisco

2b

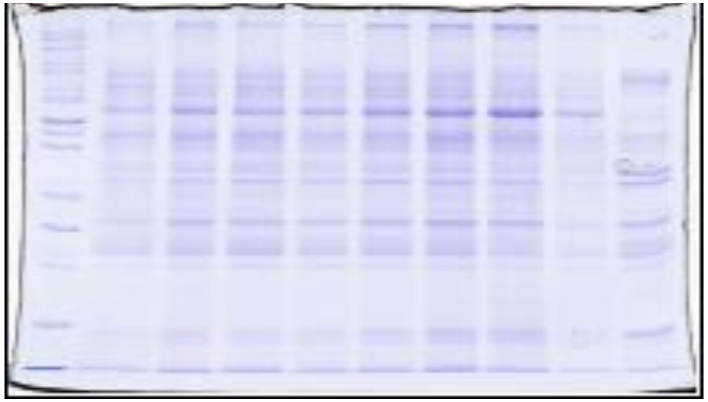

3

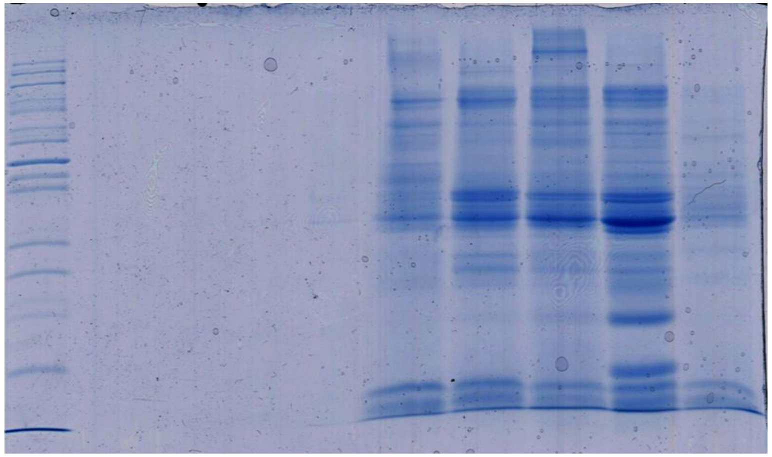

3b

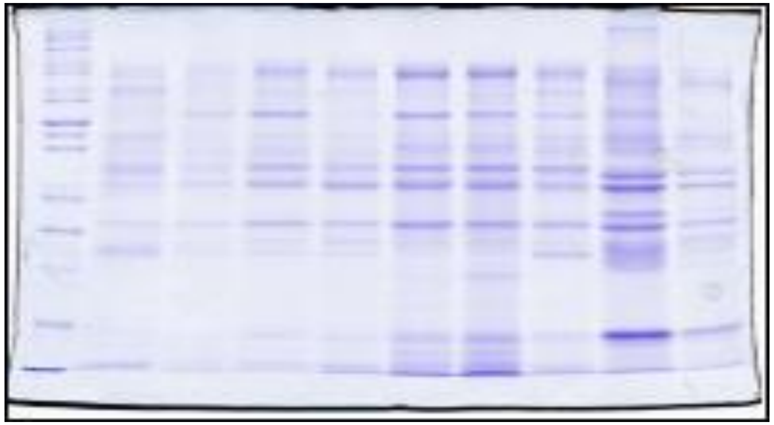

4

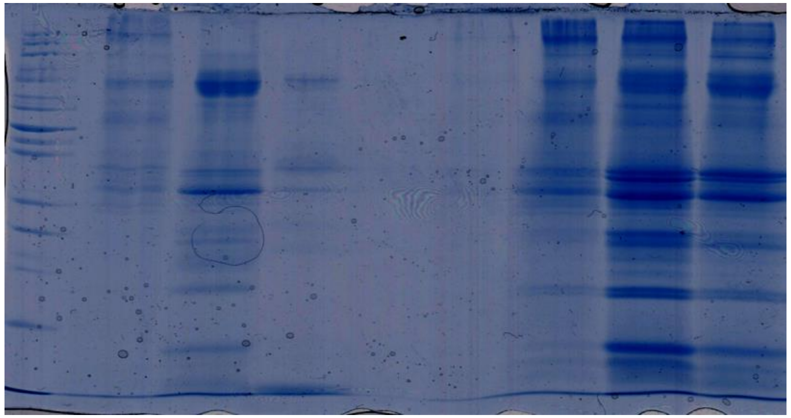

4b

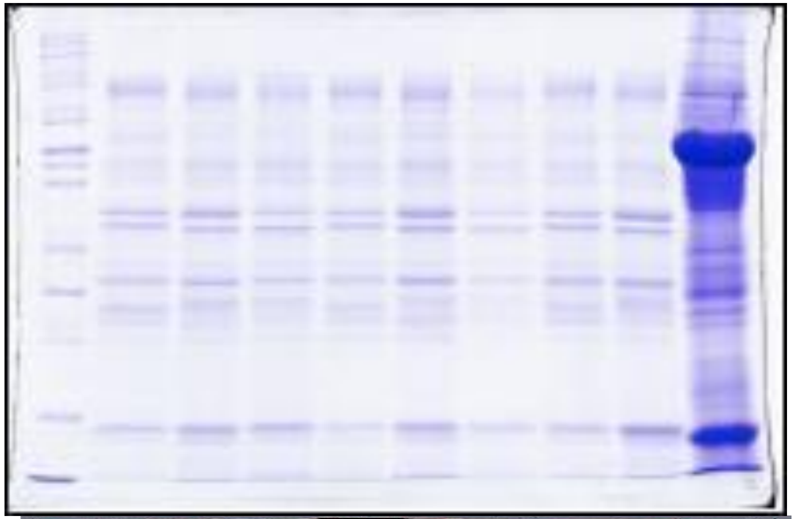

5

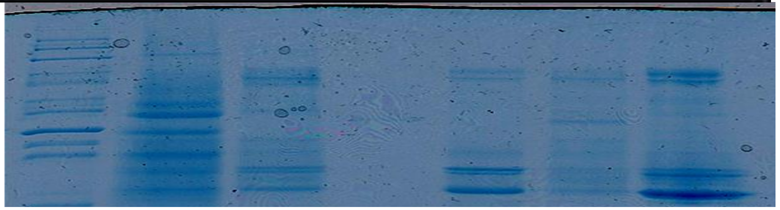

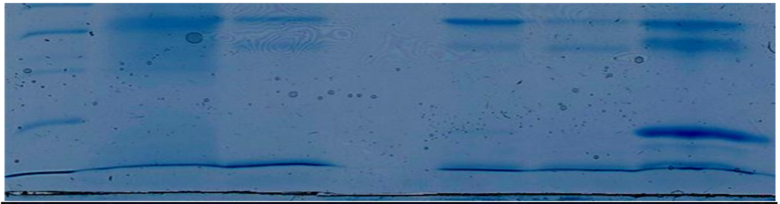

5b

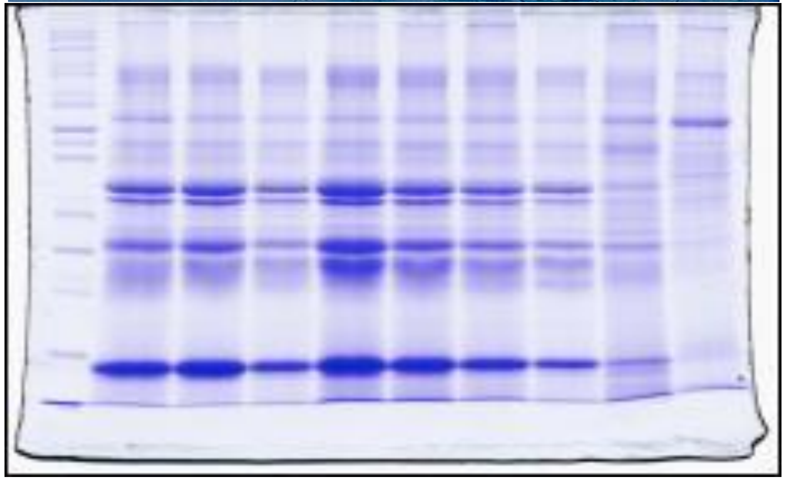

6

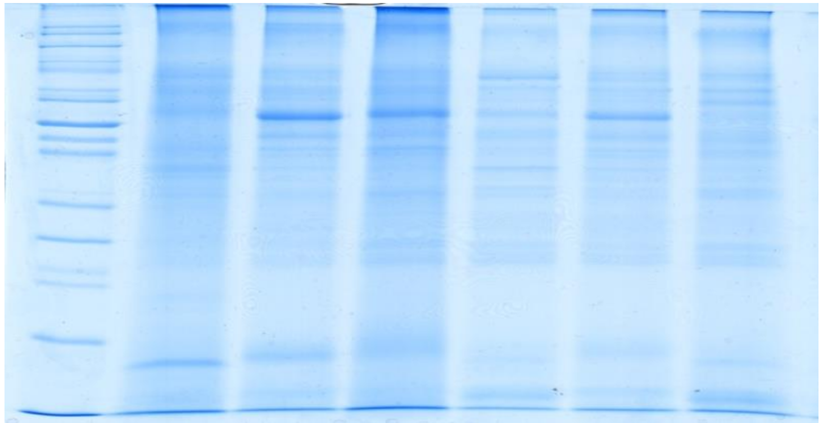

6b

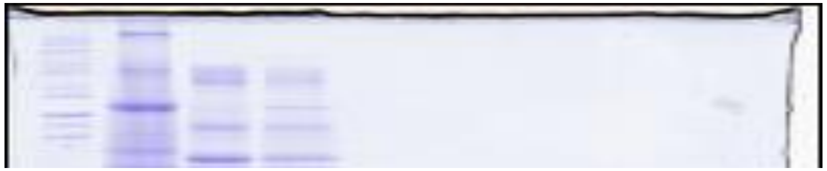

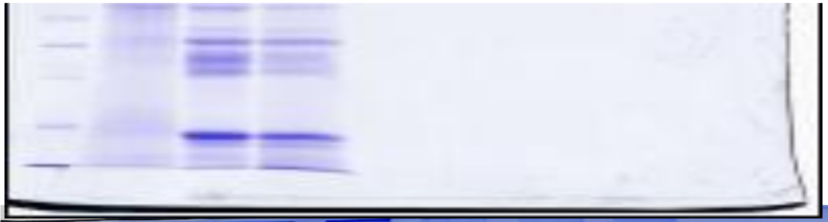

7

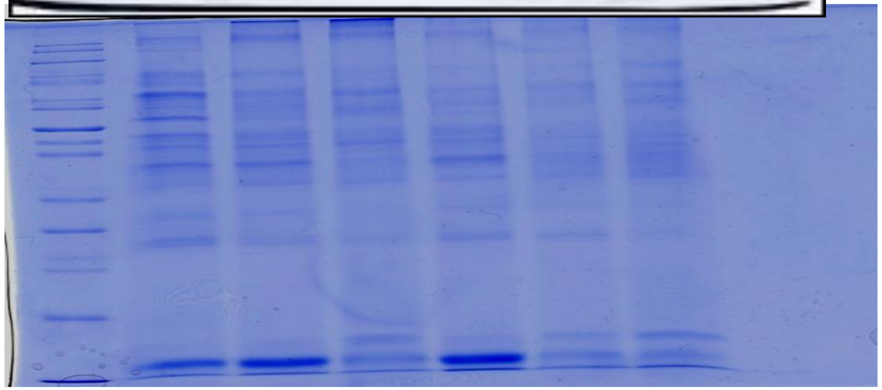

8

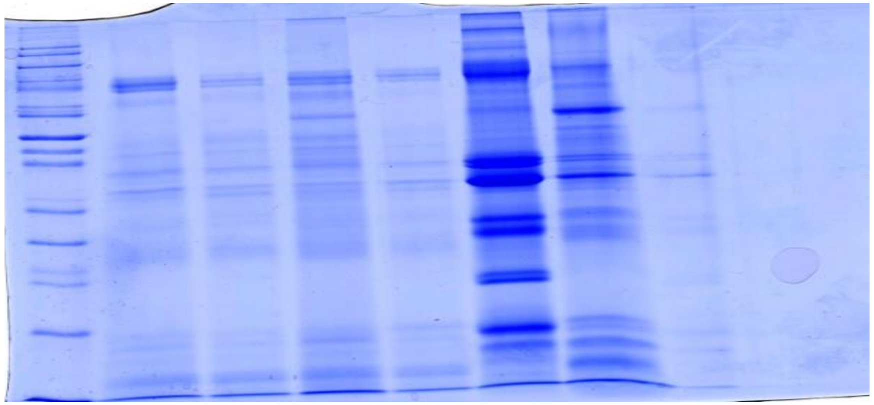

9

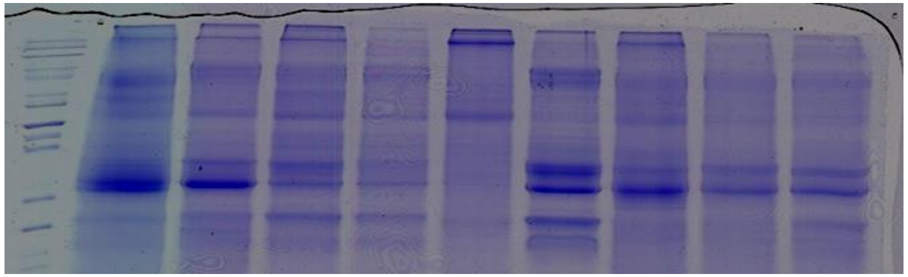

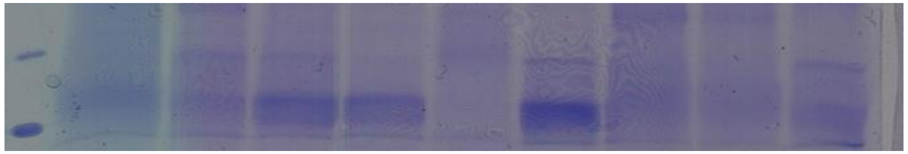

10

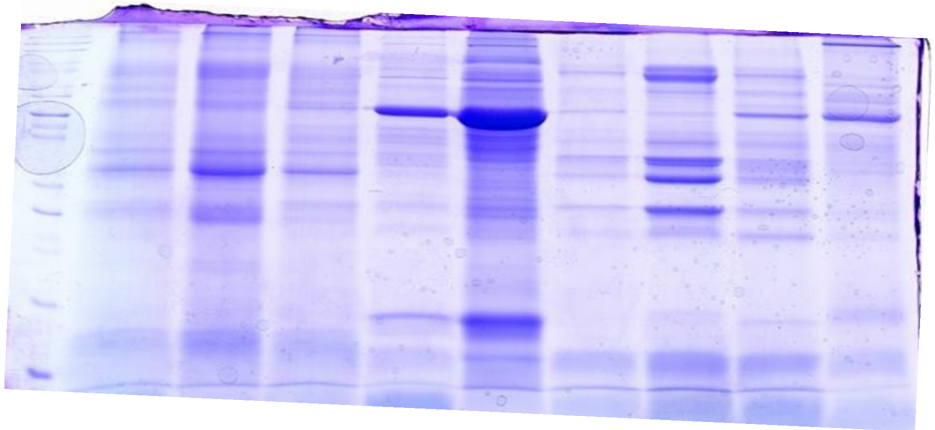

13

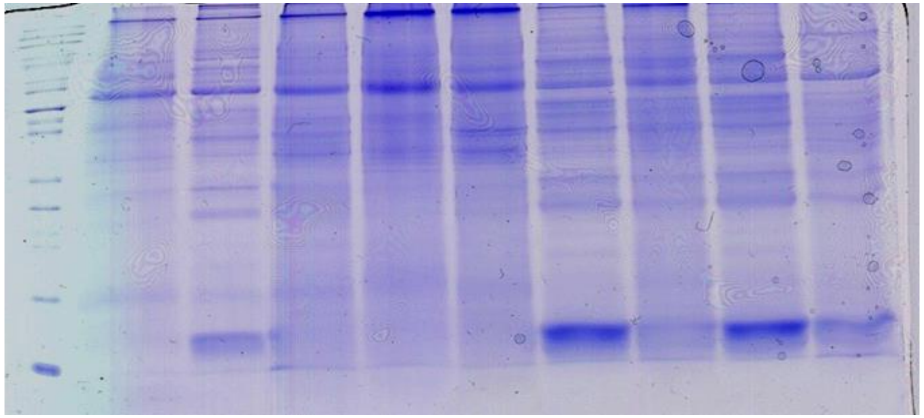

14

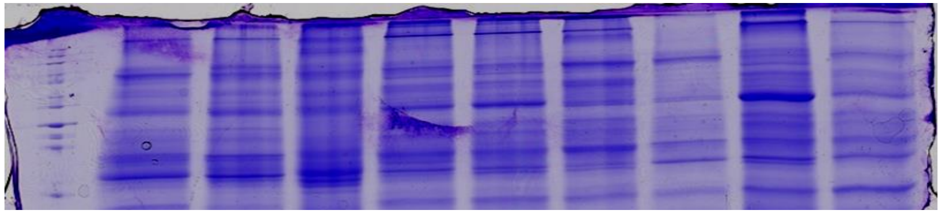

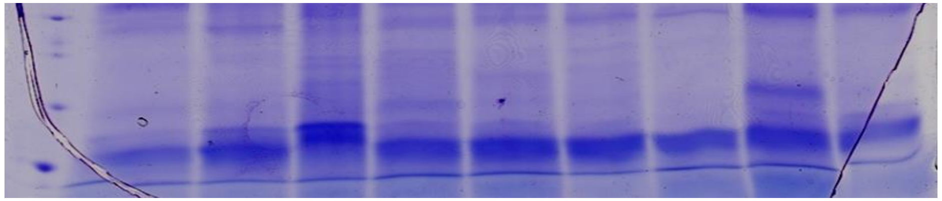

15

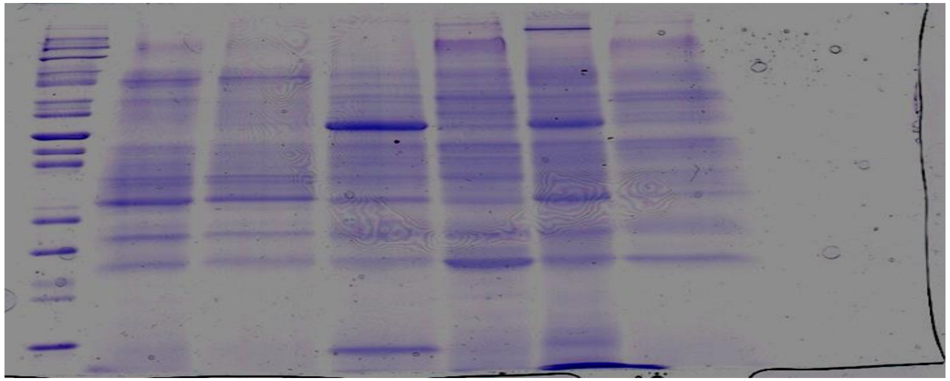

16

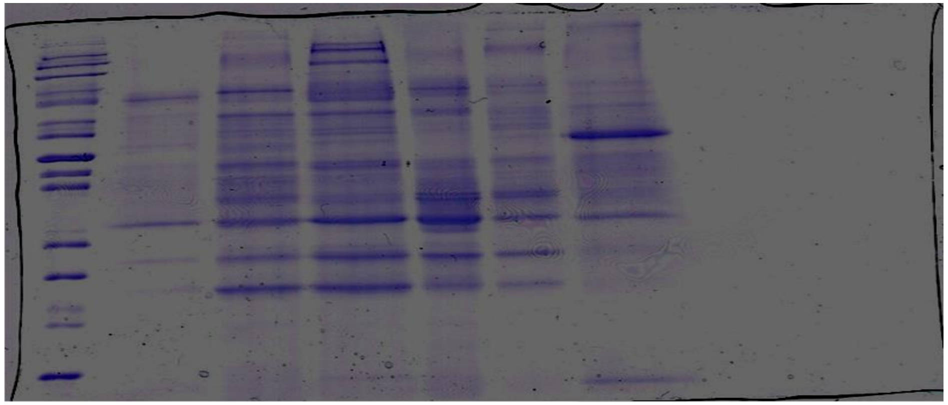

17

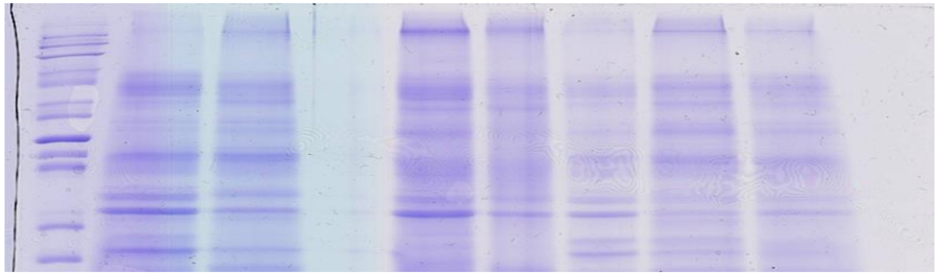

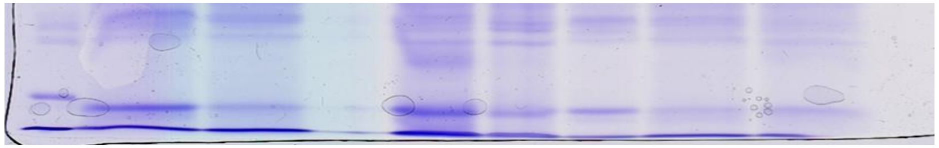

18

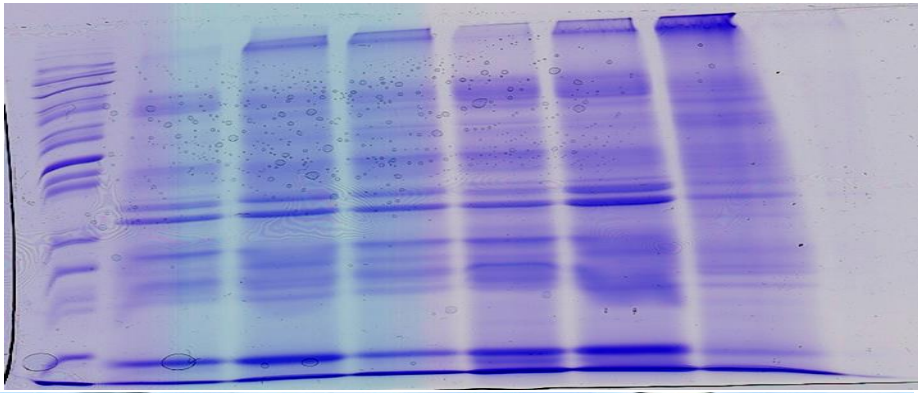

19

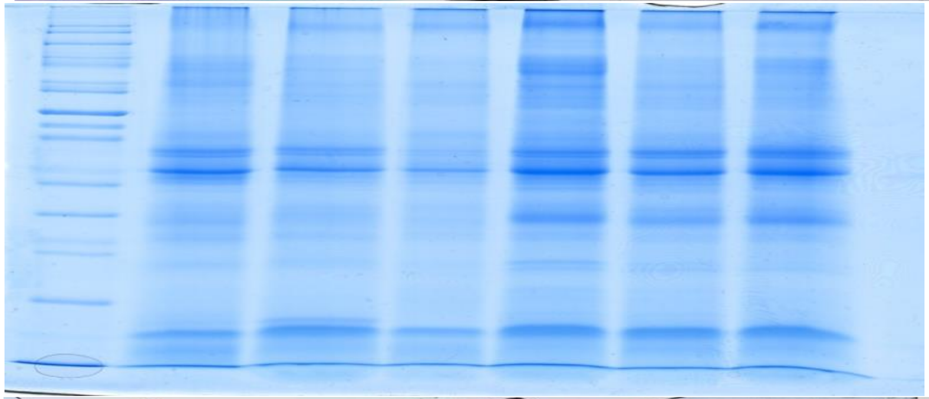

20

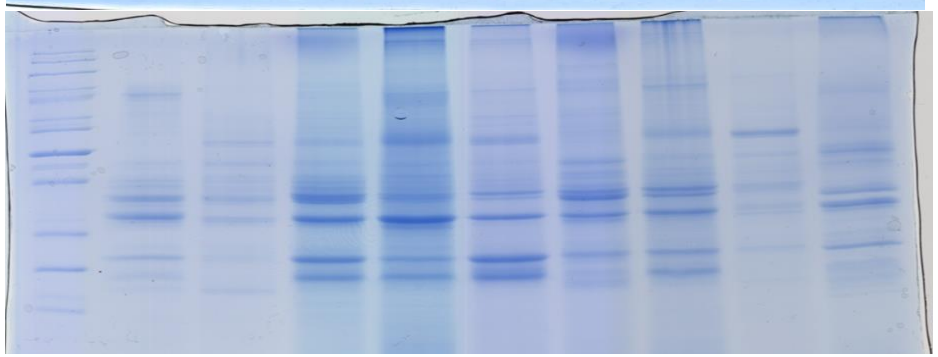

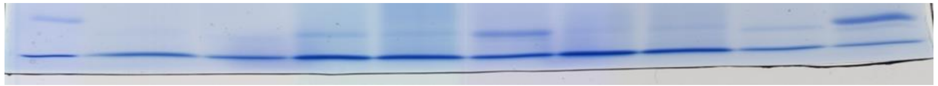

21
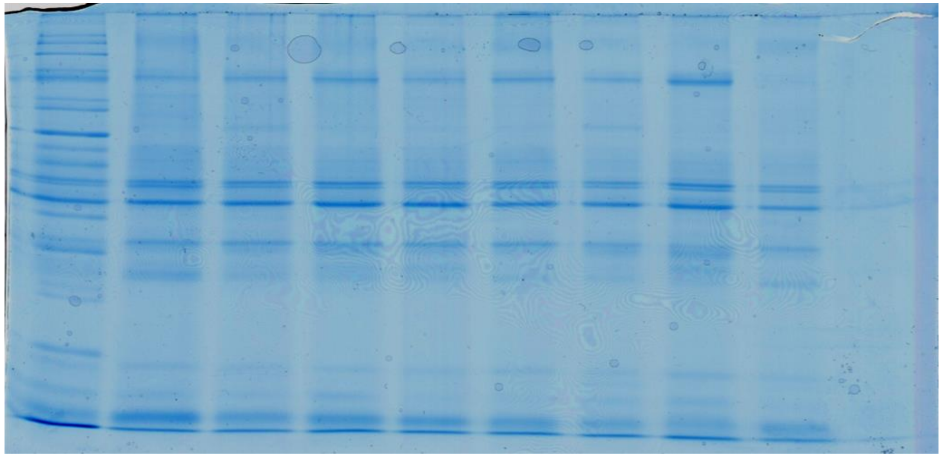

22
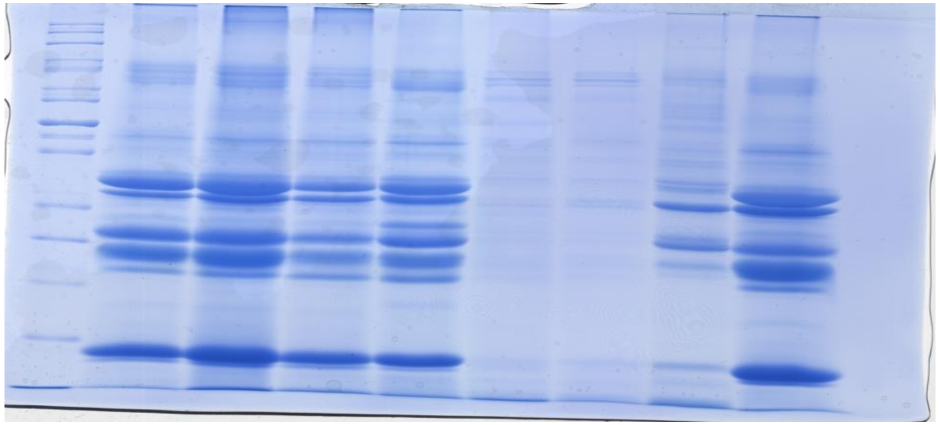

23
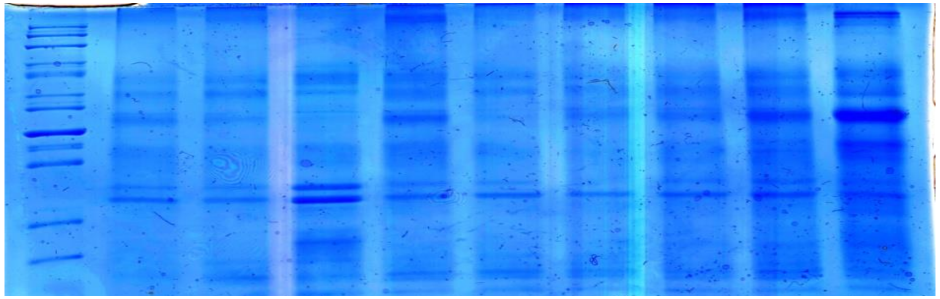

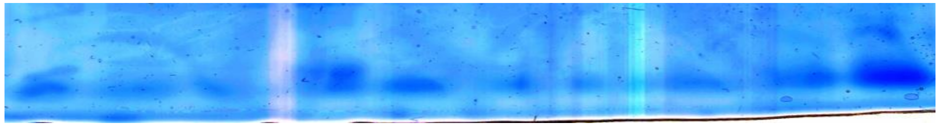

24

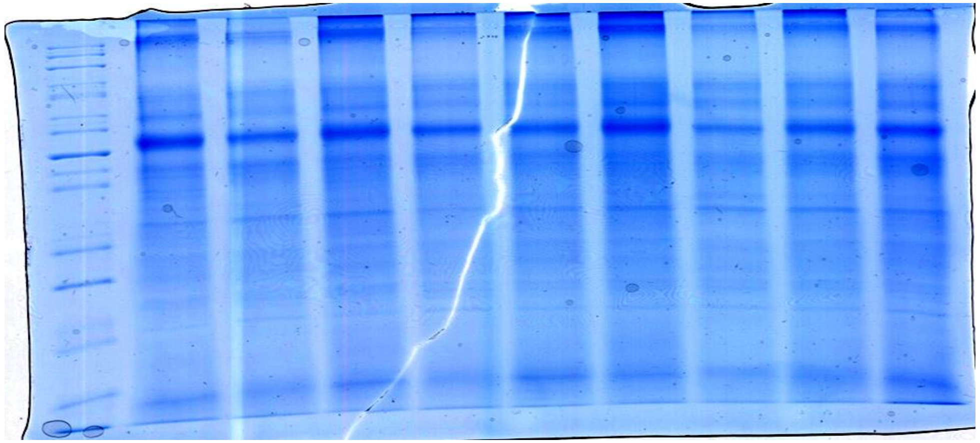

25

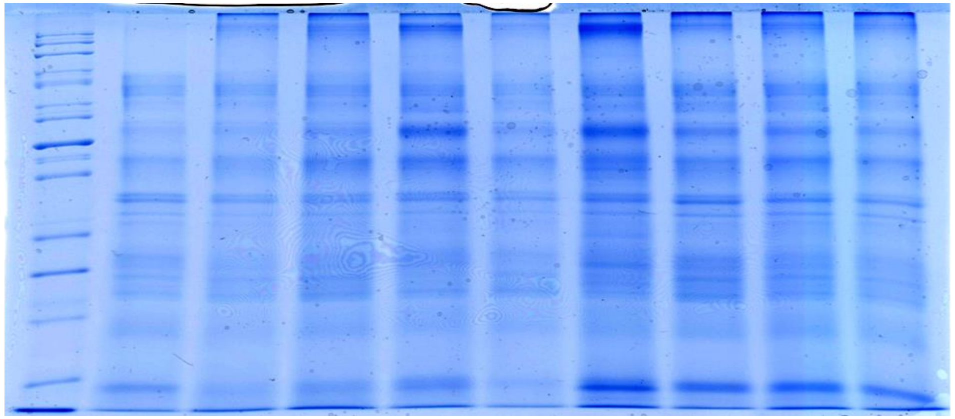

27

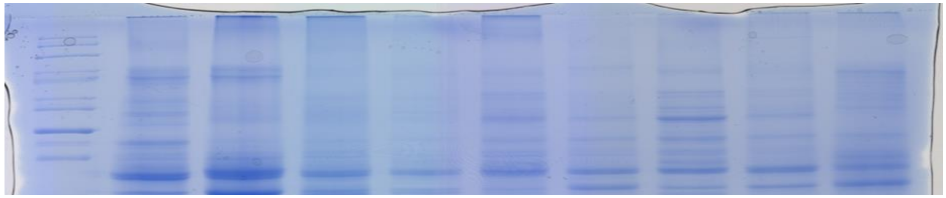

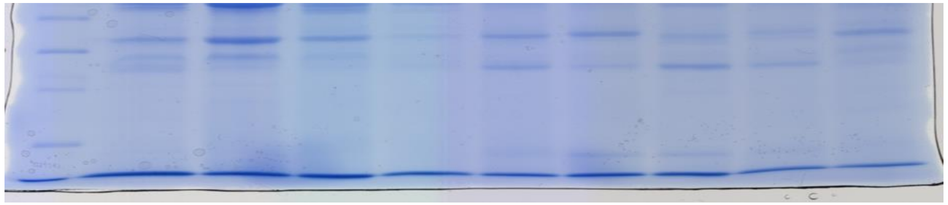

28

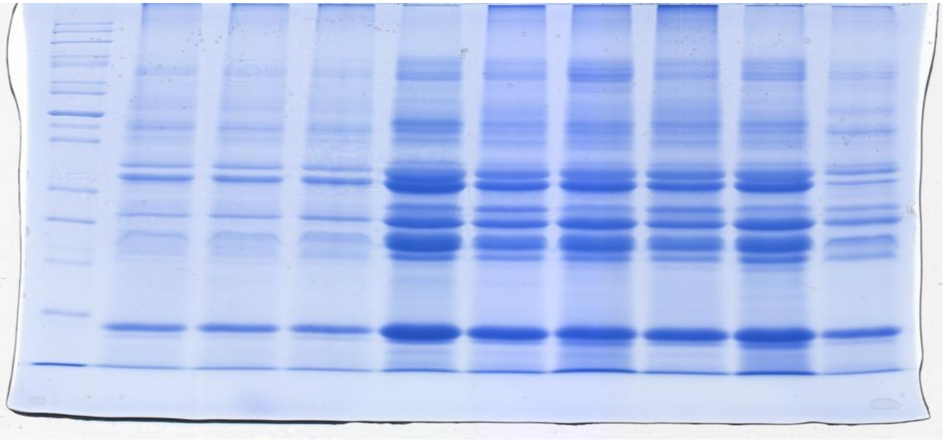

29

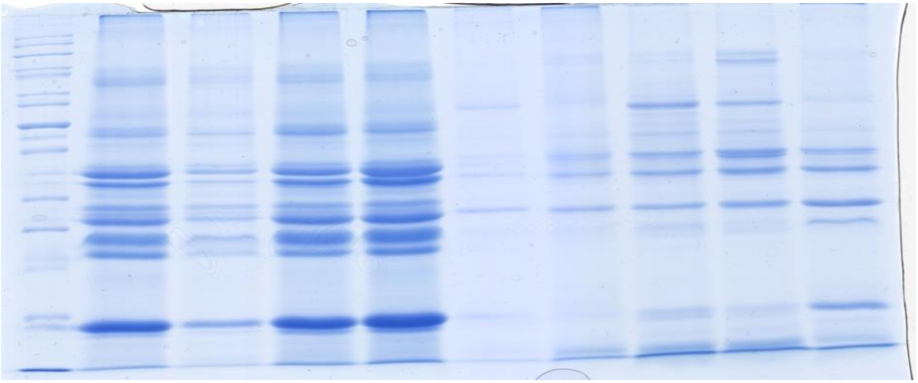

30

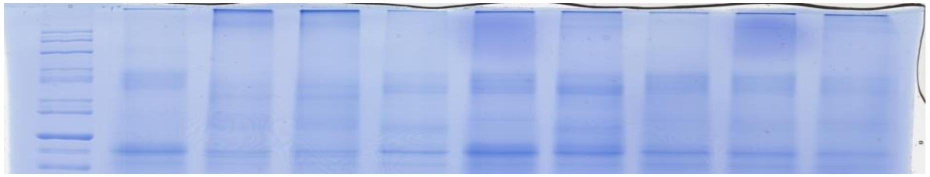

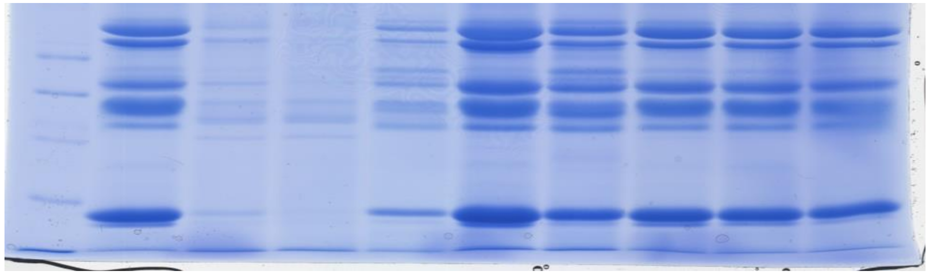

31

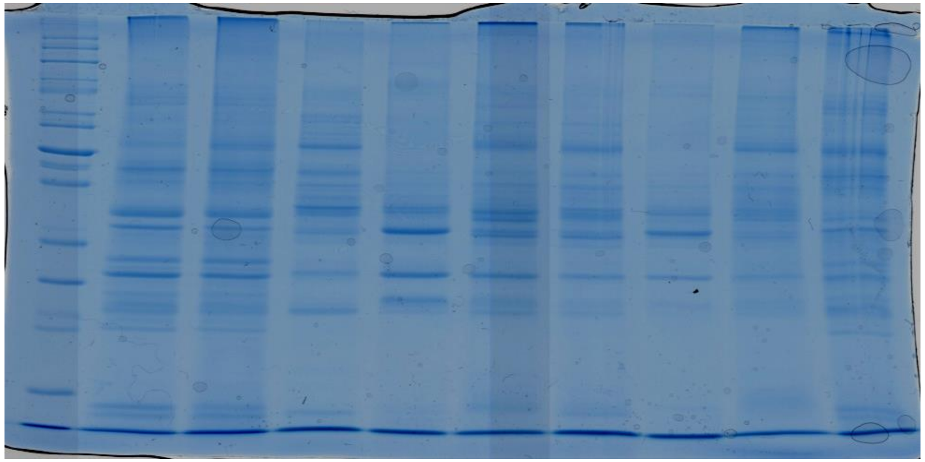

32

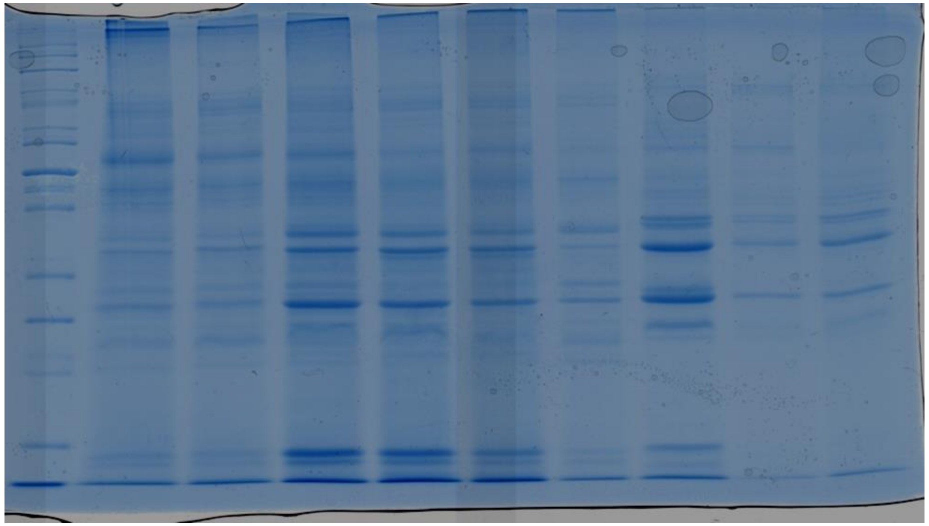

33

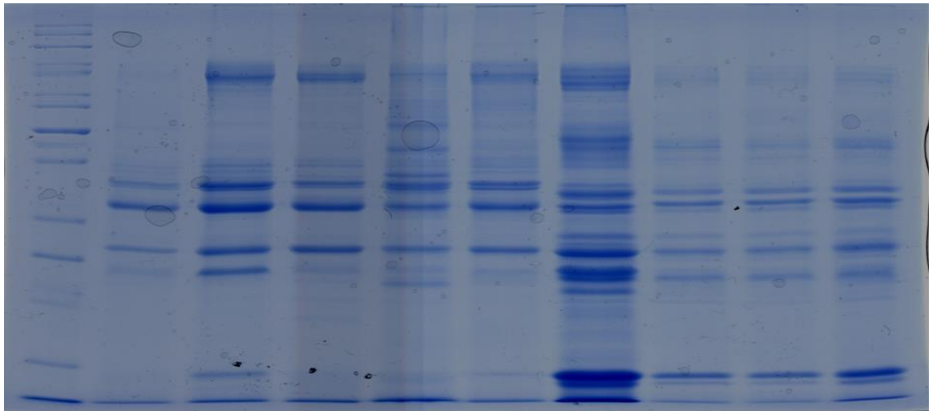

34

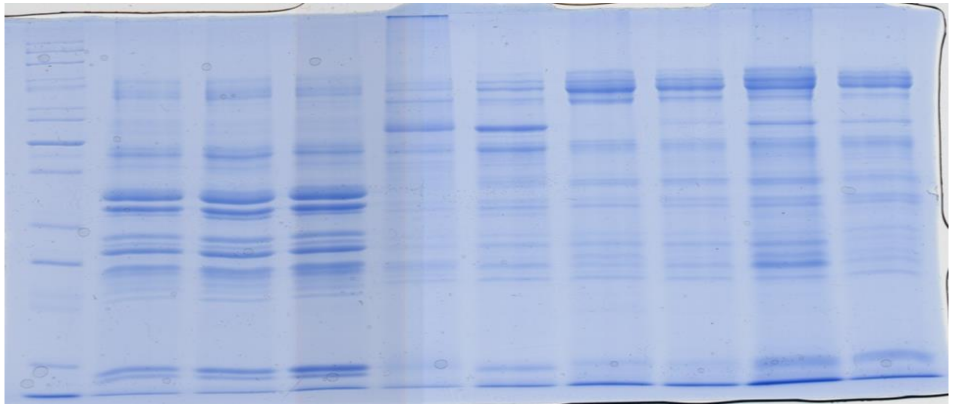

35

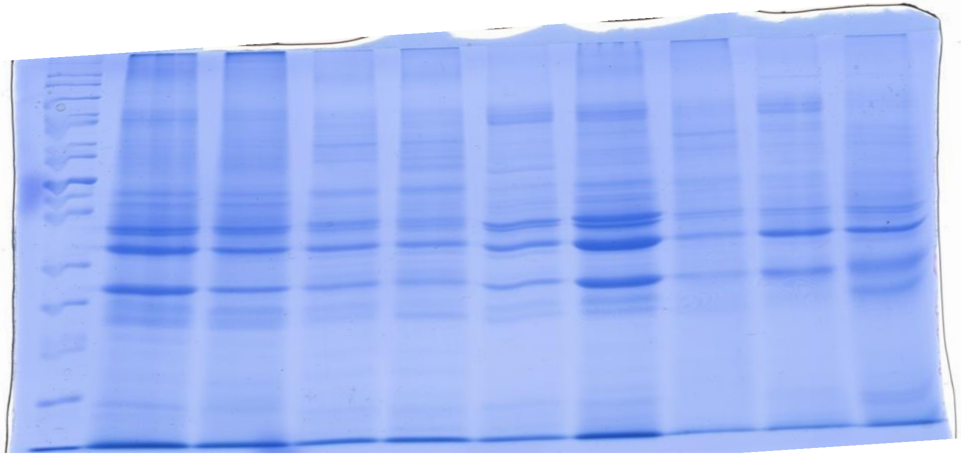

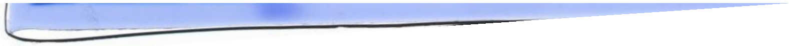

36

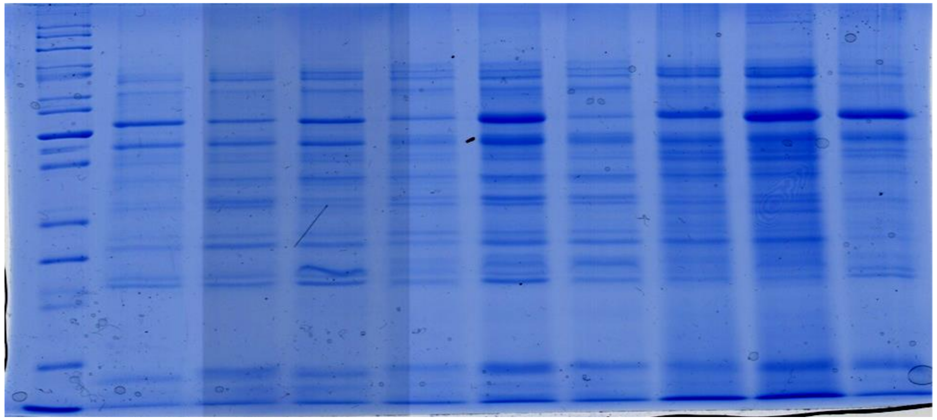

37

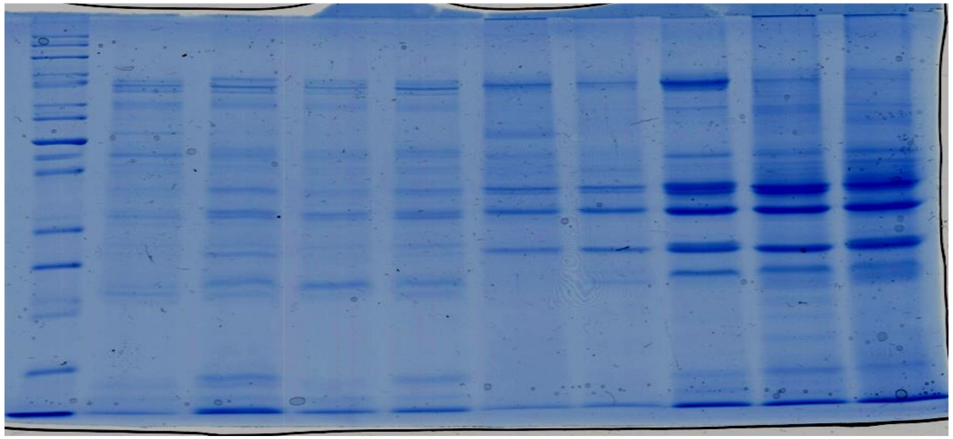

38

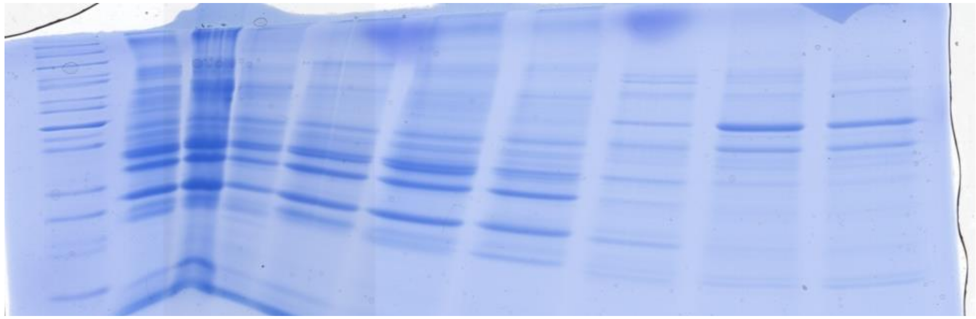

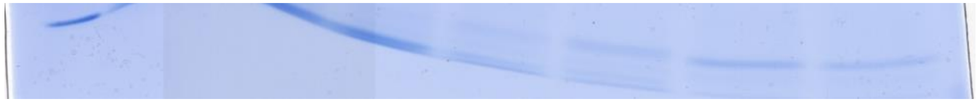

Supplement: S2 Dataset — (PDF) [file pone.0207253.s004.pdf]
