# Supplementary material for: Plant immunity in natural populations and agricultural fields: Low presence of pathogenesis-related proteins in Solanum leaves
Source: PLoS One. 2018 Nov 9;13(11):e0207253. doi: 10.1371/journal.pone.0207253 (PMC6226184; doi:10.1371/journal.pone.0207253)

Solanum species	Clone	Site	Date	Treatment	Sample id	Gel id	Lane id	PRI 10kDa	PR2+3 10-30kDa
					ladder	1:1	1		
S. dulcamara		Alnarp hedge	2012-06-08		1-3	1:1	2	0	0
S. dulcamara		Alnarp hedge	2012-06-08		4-6+25	1:1	3	0	1
S. dulcamara		Alnarp hedge	2012-06-08		7-9	1:1	4	0	0
S. dulcamara		Alnarp hedge	2012-06-08		10-12	1:1	5	0	1
S. dulcamara		Alnarp hedge	2012-06-08		13-15+26	1:1	6	0	1
S. dulcamara		Alnarp hedge	2012-06-08		16-17	1:1	7	0	0
S. dulcamara		Alnarp hedge	2012-06-08		19-21	1:1	8	0	0
S. dulcamara		Alnarp hedge	2012-06-08		22-24	1:1	9	0	0
S. dulcamara		Lomma 1	2012-06-19		53-55	1:1	10	0	0
					ladder	1:2	1		
S. dulcamara		Lomma 1	2012-06-19		56-58	1:2	2	0	1
S. dulcamara		Lomma 1	2012-06-19		62-64	1:2	3	0	0
S. dulcamara		Lomma 1	2012-06-19		65-68	1:2	4	1	1
S. dulcamara		Lomma 1	2012-06-19		69-71	1:2	5	0	0
S. dulcamara		Lomma 1	2012-06-19		72-74	1:2	6	1	1
S. dulcamara		Lomma 1	2012-06-19		75-77	1:2	7	0	0
S. dulcamara		Lomma 2	2012-06-19		85-87	1:2	8	0	0
S. dulcamara		Lomma 2	2012-06-19		88-90	1:2	9	0	0
S. dulcamara		Lomma 2	2012-06-19		91-93	1:2	10	0	0
					ladder	1:3	1		
S. dulcamara		Lomma 2	2012-06-19		94-96	1:3	2	0	0
S. dulcamara		Lomma 2	2012-06-19		97-99	1:3	3	0	0
S. dulcamara		Lomma 2	2012-06-19		100-102+109	1:3	4	N/A	0
S. dulcamara		Lomma 2	2012-06-19		103-105+110	1:3	5	0	0
S. dulcamara		Lomma 2	2012-06-19		106-108	1:3	6	0	0
S. dulcamara		Lomma 3	2012-06-29		117-119+147	1:3	7	0	1
S. dulcamara		Lomma 3	2012-06-29		120-122+148	1:3	8	0	0

S. dulcamara	Lomma 3	2012-06-29	123-125+149	1:3	9	N/A	0
S. dulcamara	Lomma 3	2012-06-29	126-128+150	1:3	10	1	1
			ladder	1:4	1		
S. dulcamara	Lomma 3	2012-06-29	129-131+151	1:4	2	0	0
S. dulcamara	Lomma 3	2012-06-29	132-134+152	1:4	3	0	0
S. dulcamara	Lomma 3	2012-06-29	135-137+153	1:4	4	0	0
S. dulcamara	Lomma 3	2012-06-29	138-140+154	1:4	5	0	0
S. dulcamara	Lund genetikum	2012-06-25	141-143	1:4	6	0	1
S. dulcamara	Lund genetikum	2012-06-25	157-159	1:4	7	1	1
S. dulcamara	Lund genetikum	2012-06-25	160-162	1:4	8	0	0
S. dulcamara	Lund genetikum	2012-06-25	163-165	1:4	9	0	1
S. dulcamara	Lund genetikum	2012-06-25	166-168	1:4	10	0	1
			ladder	1:5	1		
S. dulcamara	Alnarp hedge	2012-07-02	217-219	1:5	2	0	1
S. dulcamara	Alnarp hedge	2012-07-02	220-222	1:5	3	0	0
S. dulcamara	Alnarp hedge	2012-07-02	223-225	1:5	4	0	0
S. dulcamara	Alnarp hedge	2012-07-02	226-228	1:5	5	0	0
S. dulcamara	Alnarp hedge	2012-07-02	229-231	1:5	6	0	0
S. dulcamara	Alnarp hedge	2012-07-02	232-234	1:5	7	0	1
S. dulcamara	Alnarp hedge	2012-07-02	235-237	1:5	8	0	0
S. dulcamara	Alnarp hedge	2012-07-02	238-240	1:5	9	0	0
S. dulcamara	Lomma 1	2012-07-17	273-276+275a	1:5	10	0	0
			ladder	1:6	1		
S. dulcamara	Lomma 1	2012-07-17	276-278	1:6	2	0	0
S. dulcamara	Lomma 1	2012-07-17	279-281+281a	1:6	3	0	0
S. dulcamara	Lomma 1	2012-07-17	282-285	1:6	4	0	0
S. dulcamara	Lomma 1	2012-07-17	286-289	1:6	5	0	1
S. dulcamara	Lomma 1	2012-07-17	293-295	1:6	6	0	1

S. dulcamara	Lomma 1	2012-07-17	296-298	1:6	7	0	0
S. dulcamara	Lomma 2	2012-07-17	305-307	1:6	8	0	0
S. dulcamara	Lomma 2	2012-07-17	308-310	1:6	9	0	0
S. dulcamara	Lomma 2	2012-07-17	311-313	1:6	10	0	0
			ladder	1:7	1		
S. dulcamara	Lomma 2	2012-07-17	314-316	1:7	2	0	0
S. dulcamara	Lomma 2	2012-07-17	317-319	1:7	3	0	0
S. dulcamara	Lomma 2	2012-07-17	320-322	1:7	4	0	0
S. dulcamara	Lomma 2	2012-07-17	323-325	1:7	5	0	0
S. dulcamara	Lomma 2	2012-07-17	326-328	1:7	6	0	0
S. dulcamara	Lomma 3	2012-07-17	337-339	1:7	7	0	0
S. dulcamara	Lomma 3	2012-07-17	340-342+342a	1:7	8	0	0
S. dulcamara	Lomma 3	2012-07-17	343-345+345a	1:7	9	0	0
S. dulcamara	Lomma 3	2012-07-17	246-348	1:7	10	0	1
			ladder	1:8	1		
S. dulcamara	Lomma 3	2012-07-17	349-352	1:8	2	0	0
S. dulcamara	Lomma 3	2012-07-17	353-355	1:8	3	0	N/A
S. dulcamara	Lomma 3	2012-07-17	356-358	1:8	4	1	1
S. dulcamara	Lund genetikum	2012-07-30	370-372	1:8	5	0	1
S. dulcamara	Lund genetikum	2012-07-30	373-375	1:8	6	0	0
S. dulcamara	Lund genetikum	2012-07-30	379-381	1:8	7	0	0
S. dulcamara	Lund genetikum	2012-07-30	382-384	1:8	8	0	1
S. dulcamara	Lund genetikum	2012-07-30	385-387	1:8	9	0	1
S. dulcamara	Lund genetikum	2012-07-30	388-390	1:8	10	0	0
			ladder	2:1	1		
S. nigrum	Borgeby	2012-07-09	1-3+27	2:1	2	0	0
S. nigrum	Borgeby	2012-07-09	4-6+28	2:1	3	0	N/A
S. nigrum	Borgeby	2012-07-09	7-9+25	2:1	4	0	0

S. nigrum	Borgeby	2012-07-09	10-12+29	2:1	5	0	0
S. nigrum	Borgeby	2012-07-09	13-15+30	2:1	6	0	0
S. nigrum	Borgeby	2012-07-09	16-18+26	2:1	7	0	0
S. nigrum	Borgeby	2012-07-09	19+21+31	2:1	8	0	0
S. nigrum	Borgeby	2012-07-09	22-24+32	2:1	9	0	0
S. dulcamara	Alnarp hedge	2012-08-08	558-560	2:1	10	0	1
			ladder	2:2	1		
S. dulcamara	Alnarp hedge	2012-08-08	561-563	2:2	2	0	0
S. dulcamara	Alnarp hedge	2012-08-08	564-566	2:2	3	0	0
S. dulcamara	Alnarp hedge	2012-08-08	567-569	2:2	4	0	0
S. dulcamara	Alnarp hedge	2012-08-08	570-572	2:2	5	0	0
S. dulcamara	Alnarp hedge	2012-08-08	573-575	2:2	6	0	0
S. dulcamara	Alnarp hedge	2012-08-08	579-581	2:2	7	0	0
S. dulcamara	Lomma 1	2012-08-16	433-435	2:2	8	0	1
S. dulcamara	Lomma 1	2012-08-16	439-441	2:2	9	0	1
S. dulcamara	Lomma 2	2012-08-16	462-464	2:2	10	0	0
			ladder	2:3	1		
S. dulcamara	Lomma 2	2012-08-16	465-467	2:3	2	0	1
S. dulcamara	Lomma 2	2012-08-16	471-473	2:3	3	0	1
S. dulcamara	Lomma 2	2012-08-16	474-476	2:3	4	0	1
S. dulcamara	Lomma 2	2012-08-16	483-485	2:3	5	0	1
S. dulcamara	Lomma 3	2012-08-08	494-496	2:3	6	0	0
S. dulcamara	Lomma 3	2012-08-08	497-499+518	2:3	7	0	0
S. dulcamara	Lomma 3	2012-08-08	500-502	2:3	8	0	0
S. dulcamara	Lomma 3	2012-08-08	503-505+519	2:3	9	0	0
S. dulcamara	Lomma 3	2012-08-08	506-508	2:3	10	0	0
			ladder	2:4	1		
S. dulcamara	Lomma 3	2012-08-08	509-511	2:4	2	1	1

S. dulcamara		Lomma 3	2012-08-08		512-514	2:4	3	N/A	N/A
S. dulcamara		Lund genetikum	2012-08-22		600-602	2:4	4	0	1
S. dulcamara		Lund genetikum	2012-08-22		603-605	2:4	5	0	0
S. dulcamara		Lund genetikum	2012-08-22		606-608	2:4	6	0	0
S. dulcamara		Lund genetikum	2012-08-22		609-611	2:4	7	0	0
S. dulcamara		Lund genetikum	2012-08-22		612-614	2:4	8	0	1
S. dulcamara		Lund genetikum	2012-08-22		615-617	2:4	9	0	1
S. nigrum		Borgeby	2012-08-10		65-67+89	2:4	10	0	0
					ladder	2:5	1		
S. nigrum		Borgeby	2012-08-10		68-70+90	2:5	2	0	0
S. nigrum		Borgeby	2012-08-10		71-73+91	2:5	3	0	0
S. nigrum		Borgeby	2012-08-10		74-76	2:5	4	0	0
S. nigrum		Borgeby	2012-08-10		77-79	2:5	5	N/A	N/A
S. nigrum		Borgeby	2012-08-10		80-82+92	2:5	6	0	0
S. nigrum		Borgeby	2012-08-10		83-85+93	2:5	7	0	0
S. nigrum		Borgeby	2012-08-10		86-88	2:5	8	0	0
S. tuberosum	Bintje	Mosslunda	2012-06-26	untreated	5-8	2:5	9	0	0
S. tuberosum	Bintje	Mosslunda	2012-06-26	fungicide	9-12	2:5	10	0	0
					ladder	2:6	1		
S. tuberosum	Bintje	Mosslunda	2012-06-26	untreated	17-20	2:6	2	0	0
S. tuberosum	Bintje	Mosslunda	2012-06-26	fungicide	21-24	2:6	3	0	0
S. tuberosum	Bintje	Mosslunda	2012-06-26	fungicide	25-28	2:6	4	0	0
S. tuberosum	Bintje	Mosslunda	2012-06-26	untreated	29-32	2:6	5	0	0
S. tuberosum	Ovatio	Mosslunda	2012-06-26	fungicide	40-43	2:6	6	0	0
S. tuberosum	Ovatio	Mosslunda	2012-06-26	untreated	37-39+72	2:6	7	0	0
S. tuberosum	Ovatio	Mosslunda	2012-06-26	fungicide	52-55	2:6	8	0	0
S. tuberosum	Ovatio	Mosslunda	2012-06-26	untreated	56-59	2:6	9	0	0
S. tuberosum	Ovatio	Mosslunda	2012-06-26	fungicide	64-67	2:6	10	0	0

					ladder	2:7	1		
S. tuberosum	Ovatio	Mosslunda	2012-06-26	untreated	68-71	2:7	2	0	0
S. tuberosum	Desiree	Helgegården	2012-07-03	untreated	145-148	2:7	3	0	0
S. tuberosum	SW93-1015	Helgegården	2012-07-03	untreated	149-152	2:7	4	0	0
S. tuberosum	Sarpo Mira	Helgegården	2012-07-03	untreated	153-156	2:7	5	0	0
S. tuberosum	SW93-1015	Helgegården	2012-07-03	untreated	157-160	2:7	6	0	0
S. tuberosum	Desiree	Helgegården	2012-07-03	untreated	161-164	2:7	7	0	0
S. tuberosum	Sarpo Mira	Helgegården	2012-07-03	untreated	165-168	2:7	8	0	0
S. tuberosum	Desiree	Helgegården	2012-07-03	untreated	169-172	2:7	9	0	0
S. tuberosum	Sarpo Mira	Helgegården	2012-07-03	untreated	173-174	2:7	10	0	0
					ladder	2:8	1		
S. tuberosum	SW93-1015	Helgegården	2012-07-03	untreated	177-180	2:8	2	0	0
S. tuberosum	Desiree	Borgeby	2012-07-09	untreated	253-256	2:8	3	0	0
S. tuberosum	Desiree	Borgeby	2012-07-09	untreated	257-260	2:8	4	0	0
S. tuberosum	Desiree	Borgeby	2012-07-09	untreated	261-264	2:8	5	0	0
S. tuberosum	Sarpo Mira	Borgeby	2012-07-09	untreated	265-268	2:8	6	0	0
S. tuberosum	Sarpo Mira	Borgeby	2012-07-09	untreated	269-272	2:8	7	0	0
S. tuberosum	Sarpo Mira	Borgeby	2012-07-09	untreated	273-276	2:8	8	0	0
S. tuberosum	Bintje	Mosslunda	2012-07-26	untreated	185-188	2:8	9	0	0
S. tuberosum	Bintje	Mosslunda	2012-07-26	fungicide	189-192	2:8	10	0	0
					ladder	3:1	1		
S. tuberosum	Bintje	Mosslunda	2012-07-26	untreated	197-200	3:1	2	0	0
S. tuberosum	Bintje	Mosslunda	2012-07-26	fungicide	201-204	3:1	3	0	0
S. tuberosum	Bintje	Mosslunda	2012-07-26	fungicide	205-208	3:1	4	0	0
S. tuberosum	Bintje	Mosslunda	2012-07-26	untreated	209-212	3:1	5	0	0
S. tuberosum	Ovatio	Mosslunda	2012-07-26	fungicide	217-220	3:1	6	1	1
S. tuberosum	Ovatio	Mosslunda	2012-07-26	untreated	221-224	3:1	7	1	1
S. tuberosum	Ovatio	Mosslunda	2012-07-26	fungicide	233-236	3:1	8	1	1
S. tuberosum	Ovatio	Mosslunda	2012-07-26	untreated	237-240	3:1	9	1	1
S. tuberosum	Ovatio	Mosslunda	2012-07-26	fungicide	245-248	3:1	10	1	1

					ladder	3:2	1		
S. tuberosum	Ovatio	Mosslunda	2012-07-26	untreated	249-252	3:2	2	1	1
S. tuberosum	SW93-1015	Borgeby	2012-07-20	untreated	362-365	3:2	3	0	0
S. tuberosum	SW93-1015	Borgeby	2012-07-20	untreated	366-369	3:2	4	0	0
S. tuberosum	SW93-1015	Borgeby	2012-07-20	untreated	370-373	3:2	5	N/A	N/A
S. tuberosum	Desiree	Helgegården	2012-07-24	untreated	325-328	3:2	6	1	0
S. tuberosum	SW93-1015	Helgegården	2012-07-24	untreated	329-332	3:2	7	1	1
S. tuberosum	Sarpo Mira	Helgegården	2012-07-24	untreated	333-336	3:2	8	1	1
S. tuberosum	Sarpo Mira	Helgegården	2012-07-24	untreated	337-340	3:2	9	1	N/A
S. tuberosum	Desiree	Helgegården	2012-07-24	untreated	341-344	3:2	10	0	0
					ladder	3:3	1		
S. tuberosum	SW93-1015	Helgegården	2012-07-24	untreated	345-348	3:3	2	0	1
S. tuberosum	Desiree	Helgegården	2012-07-24	untreated	349-352	3:3	3	0	0
S. tuberosum	Sarpo Mira	Helgegården	2012-07-24	untreated	353-356	3:3	4	1	1
S. tuberosum	SW93-1015	Helgegården	2012-07-24	untreated	357-360	3:3	5	1	0
S. tuberosum	Desiree	Borgeby	2012-08-10	untreated	522-524+548a	3:3	6	1	1
S. tuberosum	Desiree	Borgeby	2012-08-10	untreated	525-527+549	3:3	7	1	1
S. tuberosum	Desiree	Borgeby	2012-08-10	untreated	528-530+550	3:3	8	1	0
S. tuberosum	Sarpo Mira	Borgeby	2012-08-10	untreated	531-533+551	3:3	9	0	0
S. tuberosum	Sarpo Mira	Borgeby	2012-08-10	untreated	534-536+552	3:3	10	1	1
					ladder	3:4	1		
S. tuberosum	Sarpo Mira	Borgeby	2012-08-10	untreated	537-539+553	3:4	2	0	1
S. tuberosum	SW93-1015	Borgeby	2012-08-10	untreated	540-542+554	3:4	3	0	1
S. tuberosum	SW93-1015	Borgeby	2012-08-10	untreated	543+545+555	3:4	4	0	1
S. tuberosum	SW93-1015	Borgeby	2012-08-10	untreated	546-548+556	3:4	5	0	0
S. tuberosum	Bintje	Mosslunda	2012-08-17	untreated	382-385	3:4	6	1	1
S. tuberosum	Bintje	Mosslunda	2012-08-17	fungicide	386-389	3:4	7	1	0
S. tuberosum	Bintje	Mosslunda	2012-08-17	untreated	394-397	3:4	8	N/A	0

S. tuberosum	Bintje	Mosslunda	2012-08-17	fungicide	398-401	3:4	9	N/A	0
					ladder	3:5	1		
S. tuberosum	Bintje	Mosslunda	2012-08-17	untreated	406-409	3:5	2	N/A	0
S. tuberosum	Ovatio	Mosslunda	2012-08-17	fungicide	414-417	3:5	3	1	1
S. tuberosum	Ovatio	Mosslunda	2012-08-17	untreated	418-421	3:5	4	1	1
S. tuberosum	Ovatio	Mosslunda	2012-08-17	fungicide	430-433	3:5	5	1	1
S. tuberosum	Ovatio	Mosslunda	2012-08-17	untreated	434-437	3:5	6	1	1
S. tuberosum	Ovatio	Mosslunda	2012-08-17	fungicide	442-445	3:5	7	1	1
S. tuberosum	Ovatio	Mosslunda	2012-08-17	untreated	446-449	3:5	8	1	1
S. tuberosum	Desiree	Helgegården	2012-08-17	untreated	557-560	3:5	9	0	1
S. tuberosum	SW93-1015	Helgegården	2012-08-17	untreated	561-564	3:5	10	0	0
					ladder	3:6	1		
S. tuberosum	Sarpo Mira	Helgegården	2012-08-17	untreated	565-568	3:6	2	1	1
S. tuberosum	SW93-1015	Helgegården	2012-08-17	untreated	575-576	3:6	3	0	0
S. tuberosum	Desiree	Helgegården	2012-08-17	untreated	577-580	3:6	4	1	1
S. tuberosum	Sarpo Mira	Helgegården	2012-08-17	untreated	569-572	3:6	5	1	1
S. tuberosum	Desiree	Helgegården	2012-08-17	untreated	581-584	3:6	6	1	1
S. tuberosum	Sarpo Mira	Helgegården	2012-08-17	untreated	585-588	3:6	7	1	1
S. tuberosum	SW93-1015	Helgegården	2012-08-17	untreated	589-592	3:6	8	0	0

Gel id

1:1

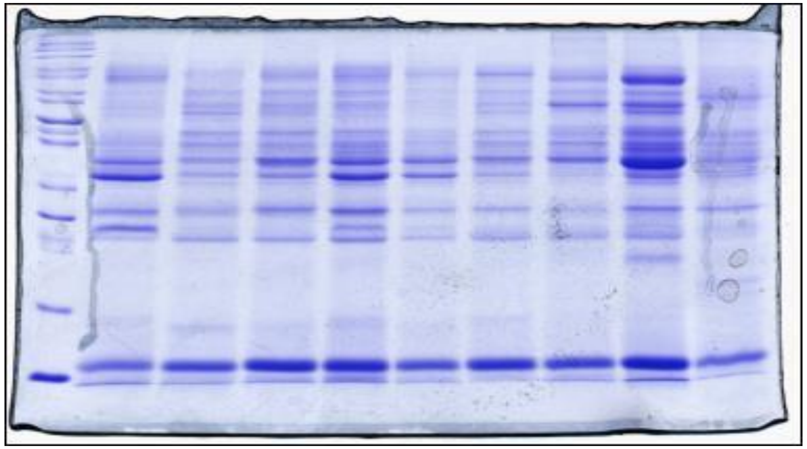

Code
0 = absence
1 = presence

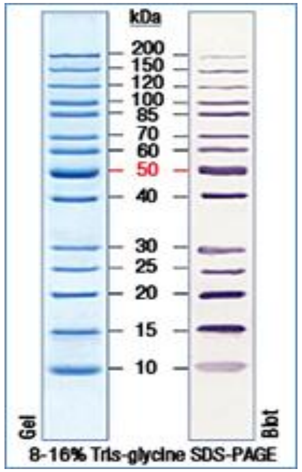

1:2

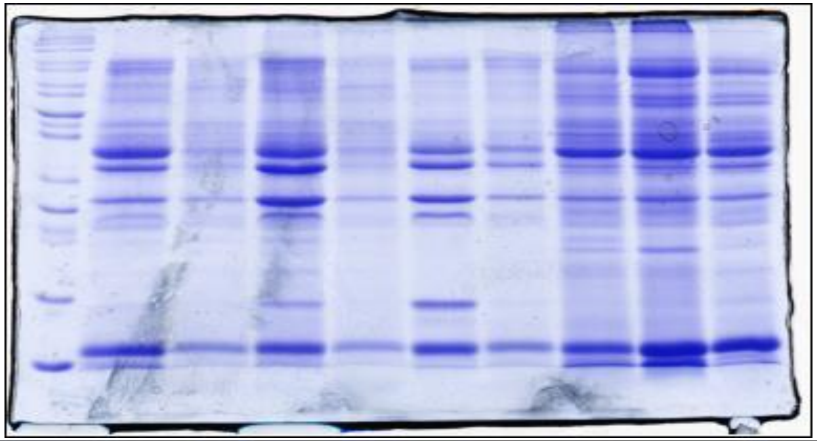

N/A = uncertain due to poor quality/presence of rubisco

1:3

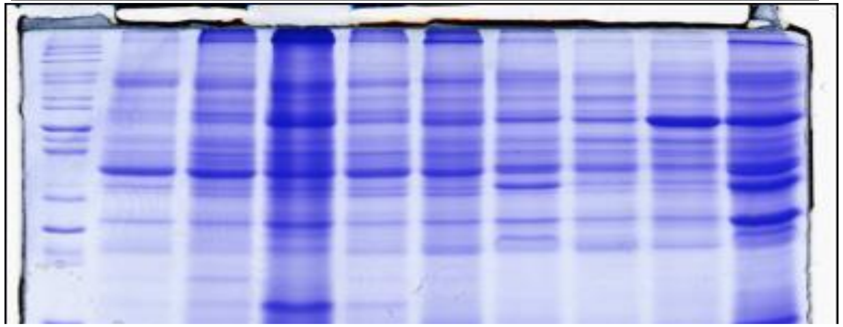

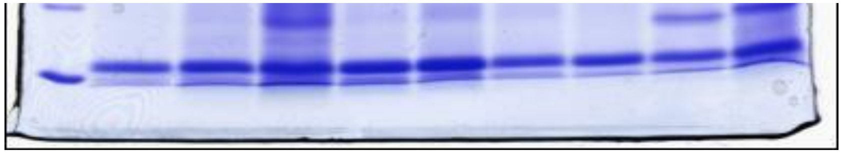

1:4

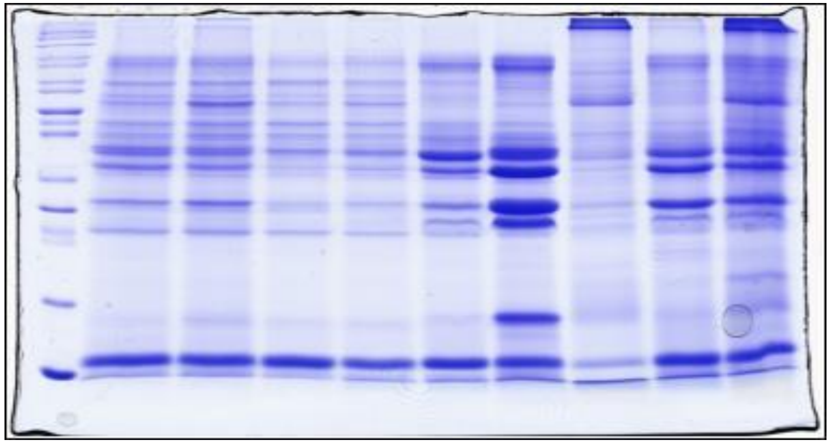

1:5

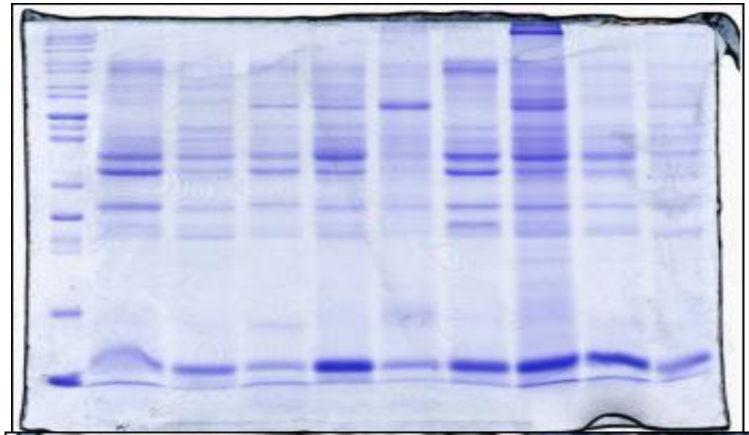

1:6

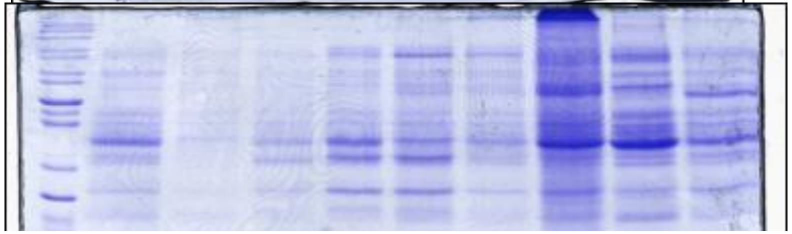

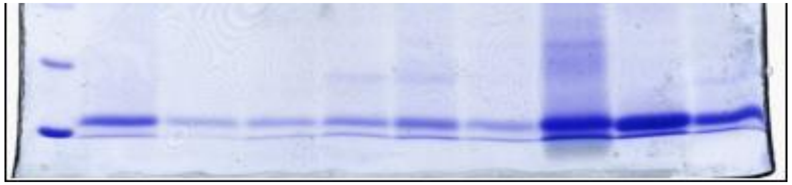

1:7

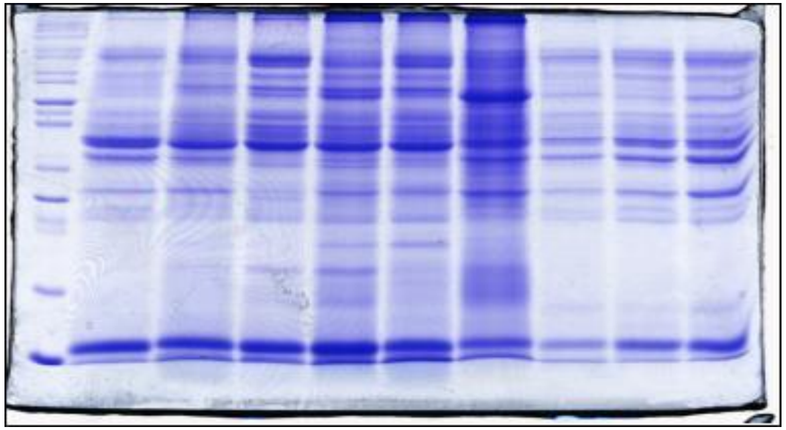

1:8

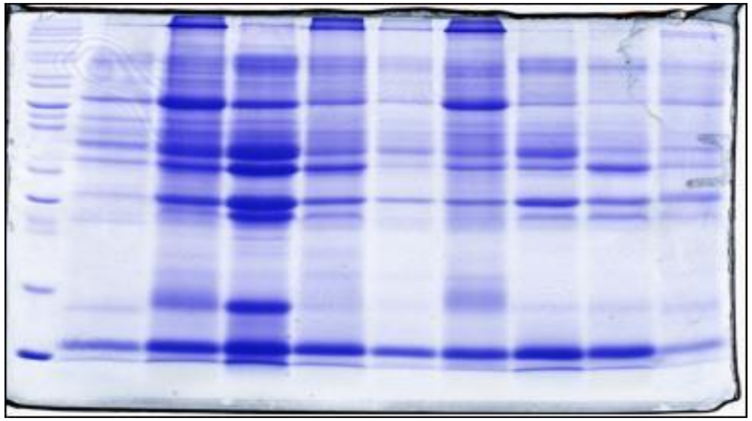

2:1

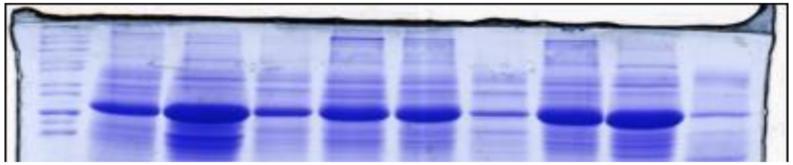

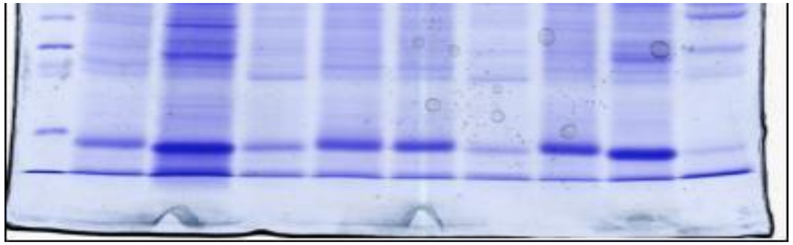

2:2

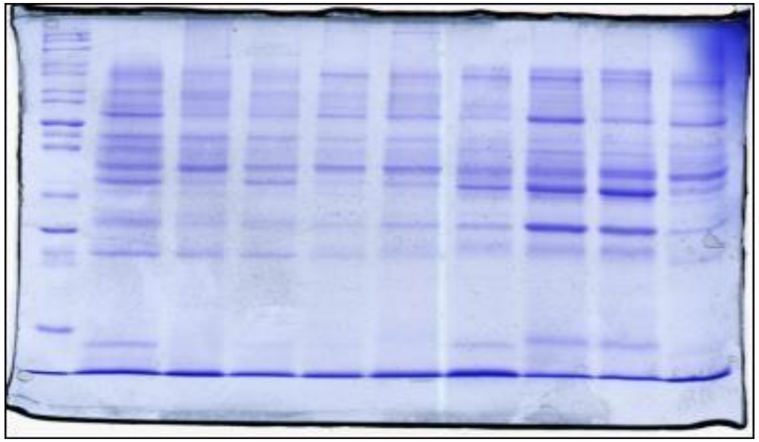

2:3

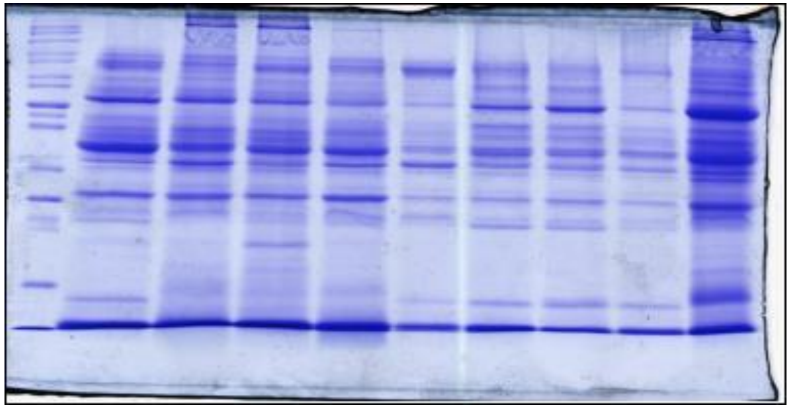

2:4

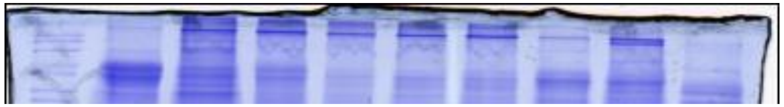

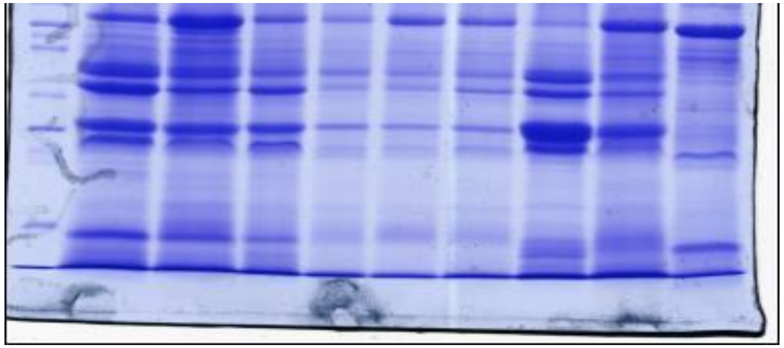

2:5

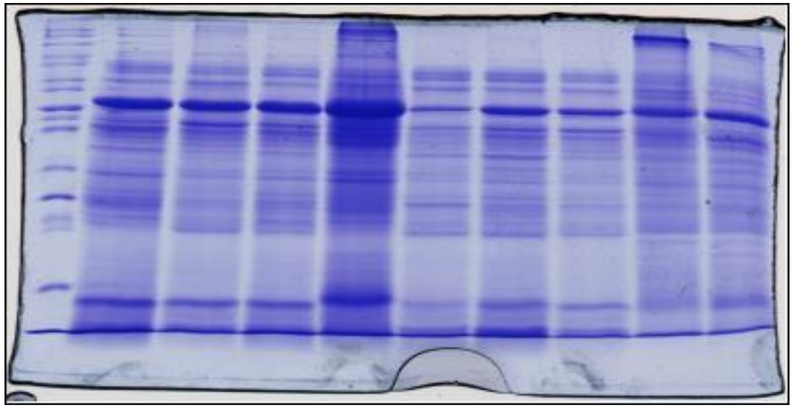

2:6

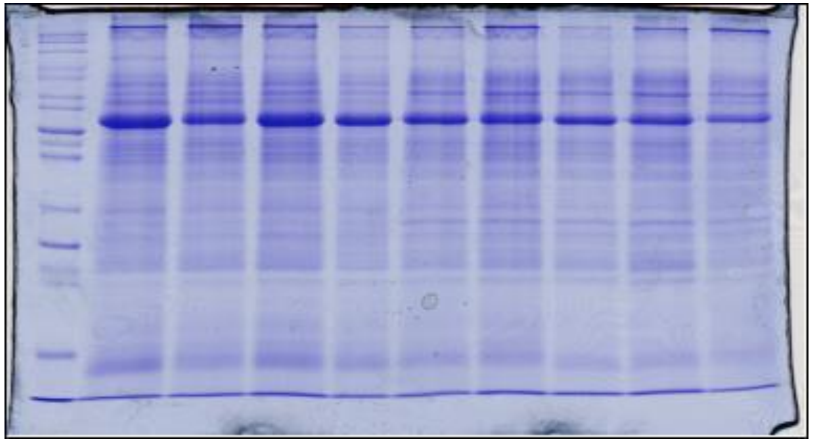

2:7

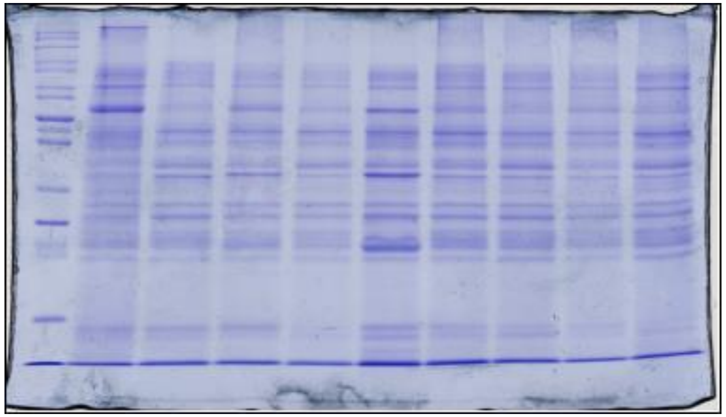

2:8

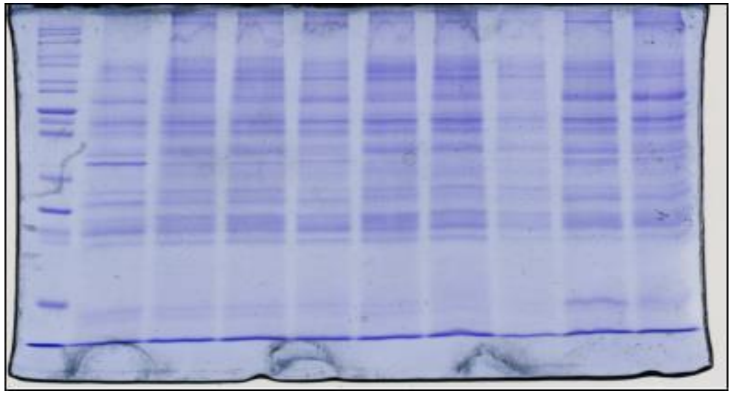

3:1

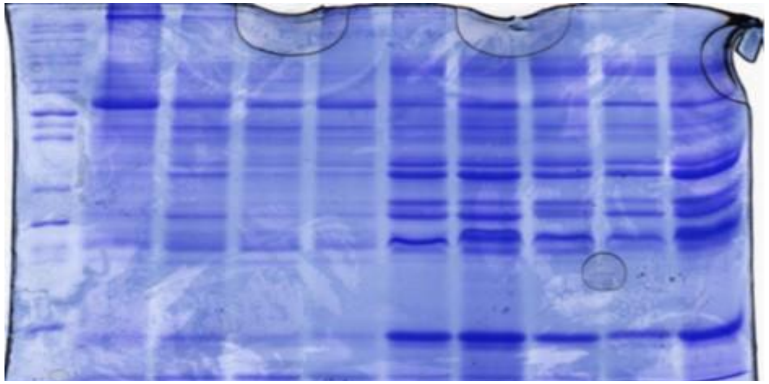

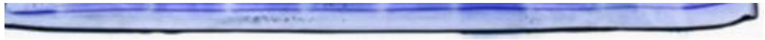

3:2

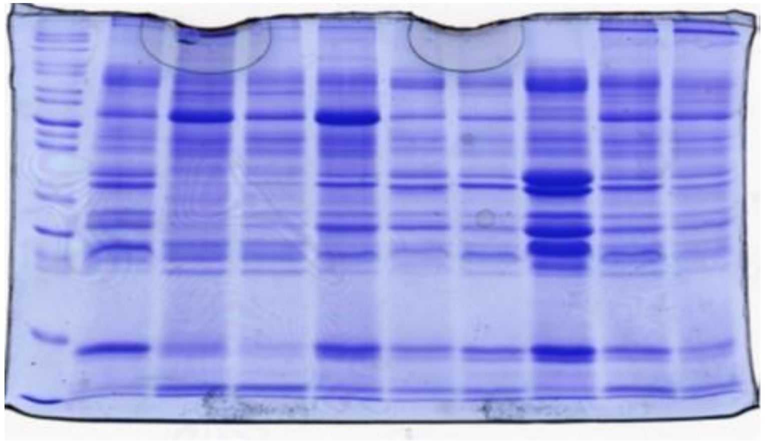

3:3

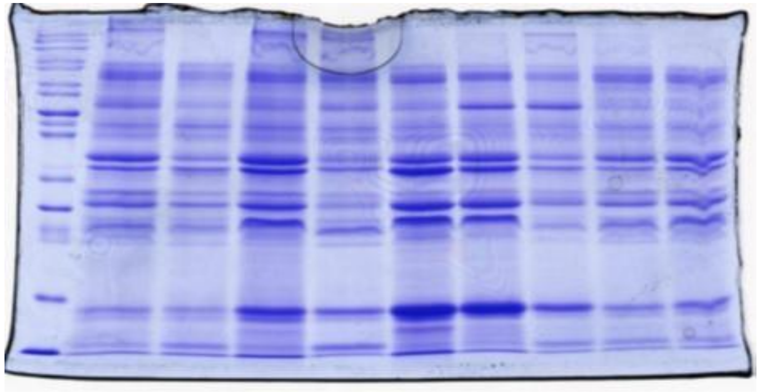

3:4

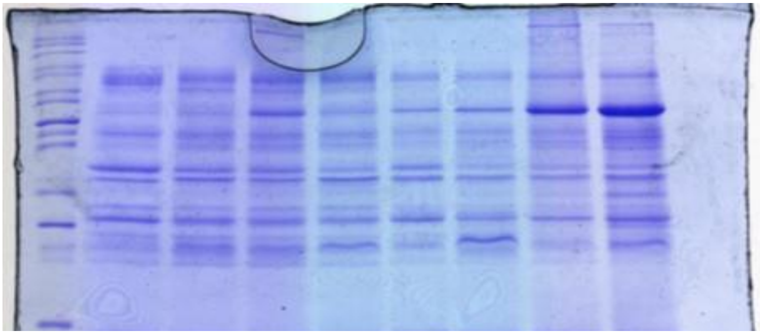

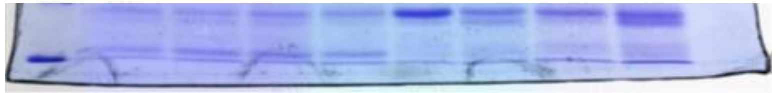

3:5

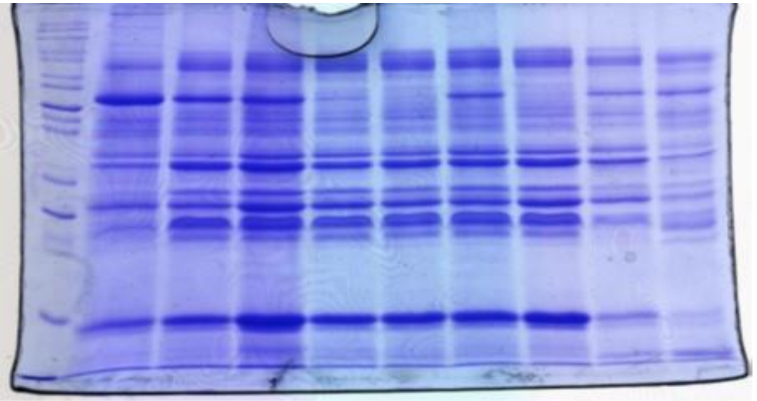

3:6

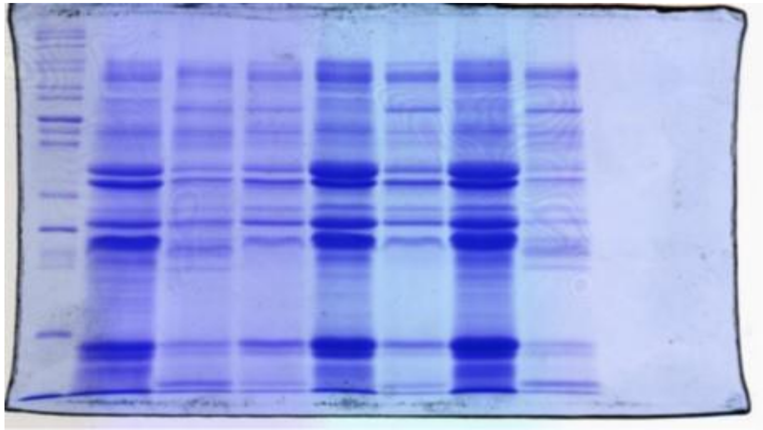

Supplement: S3 Dataset — (PDF) [file pone.0207253.s005.pdf]
